# Structural connectome and connectivity lateralization of the multimodal vestibular cortical network

**DOI:** 10.1016/j.neuroimage.2020.117247

**Published:** 2020-11-15

**Authors:** Iole Indovina, Gianfranco Bosco, Roberta Riccelli, Vincenzo Maffei, Francesco Lacquaniti, Luca Passamonti, Nicola Toschi

**Affiliations:** a Department of Biomedical and Dental Sciences and Morphofunctional Imaging, University of Messina, 98125 Messina, Italy; b Laboratory of Neuromotor Physiology, IRCCS Santa Lucia Foundation, via Ardeatina 354, 00179 Rome, Italy; c Department of Systems Medicine and Centre of Space BioMedicine, University of Rome Tor Vergata, 00173 Rome, Italy; d Department of Clinical Neurosciences, University of Cambridge, UK; e Institute of Bioimaging & Molecular Physiology, National Research Council, Milano, Italy; f IRCCS San Camillo Hospital, Venice, Italy; g Department of Biomedicine and Prevention, University of Rome “Tor Vergata”, 00133 Rome, Italy; h Department of Radiology, Athinoula A. Martinos Center for Biomedical Imaging, Boston, MA, USA

**Keywords:** Insula, MRI, HCP, OP2, PIVC, PIC, VPS

## Abstract

Unlike other sensory systems, the structural connectivity patterns of the human vestibular cortex remain a matter of debate. Based on their functional properties and hypothesized centrality within the vestibular network, the ‘core’ cortical regions of this network are thought to be areas in the posterior peri-sylvian cortex, in particular the retro-insula (previously named the posterior insular cortex-PIC), and the subregion OP2 of the parietal operculum.

To study the vestibular network, structural connectivity matrices from n=974 healthy individuals drawn from the public Human Connectome Project (HCP) repository were estimated using multi-shell diffusion-weighted data followed by probabilistic tractography and spherical-deconvolution informed filtering of tractograms in combination with subject-specific grey-matter parcellations. Weighted graph-theoretical measures, modularity, and ‘hubness’ of the multimodal vestibular network were then estimated, and a structural lateralization index was defined in order to assess the difference in fiber density of homonym regions in the right and left hemisphere. Differences in connectivity patterns between OP2 and PIC were also estimated.

We found that the bilateral intraparietal sulcus, PIC, and to a lesser degree OP2, are key ‘hub’ regions within the multimodal vestibular network. PIC and OP2 structural connectivity patterns were lateralized to the left hemisphere, while structural connectivity patterns of the posterior peri-sylvian supramarginal and superior temporal gyri were lateralized to the right hemisphere. These lateralization patterns were independent of handedness.

We also found that the structural connectivity pattern of PIC is consistent with a key role of PIC in visuo-vestibular processing and that the structural connectivity pattern of OP2 is consistent with integration of mainly vestibular somato-sensory and motor information. These results suggest an analogy between PIC and the simian visual posterior sylvian (VPS) area and OP2 and the simian parieto-insular vestibular cortex (PIVC).

Overall, these findings may provide novel insights to the current models of vestibular function, as well as to the understanding of the complexity and lateralized signs of vestibular syndromes.

## 1 Introduction

Brain areas which receive vestibular inputs are widespread across the cortical mantle and subserve complex visuo-spatial skills such as self-motion perception or spatial navigation as well as more fundamental physiological functions like postural and movement control (Lopez and Blanke, 2011). At the cortical level, vestibular inputs contribute to heading perception in visual-motion sensitive areas along the dorsal visual stream such as the occipito-temporal cortex (primarily the MT/MST complex) and superior parietal cortex (primarily the ventral intraparietal VIP) (Britten, 2008; Chen et al., 2011a; Kravitz et al., 2011). In addition, vestibular inputs which reach the posterior perisylvian regions such as the retro-insula, posterior insula, parietal opercula (OP1-4), posterior superior temporal sulcus (STS) and adjacent inferior parietal cortex (Brodmann areas 39 and 40) (Devantier et al., 2020; Mazzola et al., 2014; Shinder and Newlands, 2014) contribute to the perception of gravity and verticality (Indovina et al., 2016, 2013, 2005; Kheradmand and Winnick, 2017; Lacquaniti et al., 2014, 2013; Maffei et al., 2016; Rousseau et al., 2016; Rousseaux et al., 2015). Likewise, other areas in the ventral visual stream, in particular the posterior inferior temporal gyrus, are specialized for the recognition of gravitational visual features (Gallivan et al., 2014; Indovina et al., 2016; Maffei et al., 2015). Vestibular signals are also represented at the junction of the intraparietal sulcus with the postcentral sulcus (in an area referred to as 2v) and at the fundus of the central sulcus (area 3av) (Frank and Greenlee, 2018).

Vestibular inputs guide action initiation and movement control throughout pre-motor areas (BA 6, 44) and the frontal eye fields (BA 8) (Lopez and Blanke, 2011) and mediate spatial navigation by influencing the activity of the hippocampal formation, retro-splenial cortex, precuneus, and cingulate cortex (Hüfner et al., 2011; Indovina et al., 2016; Lopez and Blanke, 2011). However, all these areas respond to more than one sensory modality, which implies that a primary vestibular cortex does not appear to exist. In monkeys, one of the ‘core’ vestibular regions is the parieto-insular vestibular cortex (PIVC) (Chen et al., 2010; Guldin and Grüsser, 1998). Another nodal vestibular region, the visual posterior sylvian area (VPS), is located posterior to PIVC and responds to both vestibular and visual stimuli (Chen et al., 2011b). Several studies using neuroanatomical tracing techniques have characterized the pattern of connectivity of cortical vestibular areas in the monkey brain (Akbarian et al., 1994, 1993, 1992; Guldin et al., 1992; Guldin and Grüsser, 1998). In the human brain, the homologue area of the monkey PIVC is considered to be OP2 (Eickhoff et al., 2006), an area in the parietal operculum which responds to vestibular and somatosensory stimuli (Mazzola et al., 2012; zu Eulenburg et al., 2012). In contrast, the VPS homologue in humans is thought to be a region in the anterior-ventral bank of the supramarginal gyrus which responds to vestibular and visual inputs. Historically, this area has been named posterior insular cortex (PIC) (Beer et al., 2009; Frank et al., 2014; Frank and Greenlee, 2018; Sunaert et al., 1999). Due to their proximity and functional similarities, OP2 and PIC have been traditionally treated as a single region, and generically labelled as “PIVC” (Cardin and Smith, 2010; Riccelli et al., 2017). However, recent work by Frank et al. has shown that OP2 and PIC can be dissociated, at the single subject level, throughout their common response to caloric vestibular stimulation coupled with the selective response of PIC to visual object motion (Frank et al., 2016). This finding has led to renaming these structures as the PIVC+ complex (Frank and Greenlee, 2018).

Thus far, a number of studies have hypothesized a central role of the PIVC+ complex as a “hub” region mediating communication within the vestibular system (Frank et al., 2016; Frank and Greenlee, 2018; zu Eulenburg et al., 2012). Furthermore, it has been suggested that vestibular function may have hemispheric dominance, being predominantly located in the right hemisphere in right-handed individuals or in the left hemisphere in left-handed individuals (Dieterich et al., 2003; Janzen et al., 2008; Schlindwein et al., 2008). Vestibular areas are activated bilaterally by vestibular stimulation, though more in the ipsilateral hemisphere (Lopez et al., 2012; Schlindwein et al., 2008). In addition, it has been shown that in healthy right-handed individuals, right vestibular stimulation elicits higher activity of ipsilateral regions as compared to left vestibular stimulation (Dieterich et al., 2003; Fasold et al., 2002; Lopez et al., 2012). Right handed individuals also show right lateralization of structural connectivity in the upper parts of the brainstem, thalamus (Dieterich et al., 2017), and PIVC (Wirth et al., 2018). In contrast, PIC displays a trend towards left lateralization of its structural connectivity (Wirth et al., 2018). Finally, right lateralization of functional connectivity has been found in the middle posterior and inferior insula (Kirsch et al., 2018).

In this study, we investigated the structural connectivity properties of the multimodal vestibular system by using structural connectomic metrics derived from a graph-analysis theoretical framework. In essence, we tested whether distinct modules of the vestibular cortical network can be identified and whether their patterns of structural connectivity in right handers are more integrated in the right hemisphere as compared to the left hemisphere. We also assessed the role of posterior perisylvian regions (in particular OP2 and PIC) as network ‘hubs’ which implies their leading role in driving the ‘communication’ patterns between the other nodes within the network. In this context, we compared the whole-brain structural connectivity pathways of OP2 and PIC, and tested whether lateralization or hemispheric dominance of the structural connectivity patterns within the vestibular network is influenced by handedness.

## 2 Methods

### 2.1 Participants

We employed data from 974 healthy subjects (age range: 22-36 years) available in the recent S1200 Human Connectome Project (HCP) data release (see https://www.humanconnectome.org/storage/app/media/documentation/s1200/HCP_S1200_Release_Reference_Manual.pdf for full details). 794 were right handed (defined as scoring ≥ 50 in the Edinburgh inventory (Oldfield, 1971), which defines handedness scores ranging from -100 [fully left handed] to 100 [fully right handed]), while 58 were left handed (defined as scoring ≤ -50 in the Edinburgh inventory). 530 (448 right handers) were female and 444 (346 right handers) were male.

### 2.2 Magnetic Resonance Imaging (MRI) scanning

All imaging data employed in this study were acquired by the HCP consortium on a Siemens Skyra 3T scanner with a customized SC72 gradient insert. T1w 3D MPRAGE images were acquired with TR=2400 ms, TE=2.14 ms, TI=1000 ms, flip angle=8 deg, FOV=224 × 224, 0.7 mm isotropic voxel, bandwidth =210 Hz/px, iPAT=2, Acquisition time=7:40 (min:sec).

Diffusion weighted images were acquired with Spin-echo EPI sequences (b-values = 0, 1000, 2000, 3000 s/mm2 in approximately 90 gradient directions (interspersed with an approximately equal number of acquisitions on each shell). Diffusion weighting consisted of 3 shells of b=1000, 2000, and 3000 s/mm^2^. The diffusion directions were uniformly distributed directions in multiple q-space shells and optimized so that every consecutive subset of directions is also isotropic (Caruyer et al., 2013), TR=5520 ms, TE=89.5 ms, flip angle=78 deg, refocusing flip angle=160 deg, FOV=210 × 180 (RO x PE) matrix=168 × 144 (RO x PE), slice thickness =1.25 mm, 111 slices, 1.25 mm isotropic voxels, Multiband factor=3, Echo spacing=0.78 ms, BW=1488 Hz/Px, Phase partial Fourier 6/8). A full diffusion MRI session included 6 runs (approximately 9 minutes and 50 seconds each). Diffusion gradients were monopolar. Image reconstruction uses SENSE multi-channel (Sotiropoulos et al., 2013).

### 2.3 Diffusion weighted data analysis

Diffusion image preprocessing, performed by the HCP consortium, included state-of the art procedures: intensity normalization across runs, distortion correction through the ‘TOPUP’ tool (part of FSL, (Jenkinson et al., 2012)), eddy current and motion correction through the ‘EDDY’ tool (also part of FSL), gradient nonlinearity correction, calculation of resulting gradient bvalue/bvector deviation, and Registration of mean b0 to the corresponding T1w volume with FLIRT BBR+bbregister (also part of FSL). This is followed by transformation of diffusion data, gradient deviation, and gradient directions to 1.25mm structural space. Starting from these preprocessed data, anatomically constrained multi-shell, multi-tissue spherical deconvolution (Jeurissen et al., 2014) followed by probabilistic tractography and spherical-deconvolution informed filtering of tractograms (SIFT) (Smith et al., 2013) was used to estimate whole-brain tractograms (Tournier et al., 2007) in mrtrix3 (Tournier et al., 2019). We employed the probabilistic tractography by 2^nd^ order integration over fiber orientation distributions (iFOD2) algorithm (Willats et al., 2014). For each subject, 10^8^ fibers streamlines were generated and then filtered using SIFT by a factor 10 to obtain more precise anatomical correspondence in the final set of 10^7^ streamlines. Generation of all tractograms was performed on a high-performance parallel computing cluster and took approximately 80 years of single CPU time.

### 2.4 Grey matter parcellation

**Fig. 1:**
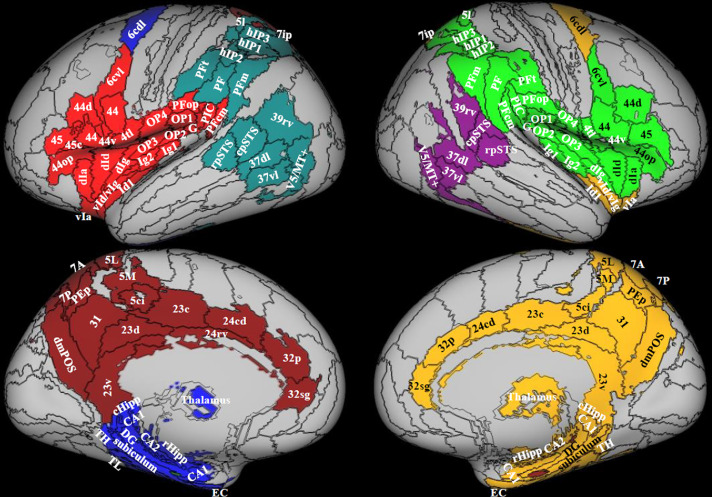
Modularity. Areas in color have been selected as belonging to the multimodal vestibular cortex. Each color represents a module, that is a cluster of areas highly connected. Data from 794 right handed individuals. There are 4 modules on the left and 3 on the right, indicating better integration of the vestibular network on the right. Regions from the composite atlas are overlapped onto the Conte69 inflated brain in workbench viewer (Glasser et al 2016).

In order to define parcels on which to base the construction of structural adjacency matrices, we followed a stepwise procedure. First, we complemented the cytoarchitectonic atlas “Anatomy” (Eickhoff et al., 2005), composed of a total of 200 regions encompassing only part of the cerebral cortex (portions of the posterior insula, cingulate, parietal, occipital and frontal cortices), the cerebellum and subcortical nuclei with the more comprehensive but less finely grained connectivity-based “Brainnetome” atlas, composed of a total of 246 regions encompassing the whole cortex and subcortical nuclei (Fan et al., 2016) in Montreal Neurological Institute (MNI space). This choice was driven by the fact that the Eickhoff atlas is the only available parcellation which includes a cytoarchitectonic parcellation of OP2 – a prominent candidate in the vestibular literature as the human homologue of PIVC. In cases where two regions from the two atlases partially overlapped, we defined two distinct regions, one by selecting the Anatomy area and one by selecting the Brainnetome area subtracted of the overlap. This resulted in an initial atlas comprised of 446 regions encompassing the whole cortex, thalamus, subcortical structures and cerebellum (see Fig. 1 for some examples). Third, given that structural connectivity estimates can depend on the volume of the involved parcels, this initial atlas was rendered symmetrical in volumes between homologous contralateral regions through 1) affine transformation to the symmetrical MNI template and 2) retaining only the intersection between each region and its contralateral homologue after flipping the atlas across the yz plane. This procedure roughly preserves the volume and architecture of each region while eliminating volume differences across contralateral homologue regions. The resulting atlas (Symmetrical atlas) was employed for lateralization and modularity analysis (see below). In addition, we created a second atlas which was designed to eliminate volume differences across seed regions. This atlas (Sphere atlas) was generated by placing spherical regions of interest (ROIs, radius: 4 mm) on the geometric center of each region of the Symmetrical atlas (See Inline Supplementary Figure 1). The Sphere atlas was employed in quantitative comparisons through graph-theoretical network measures, in which volume-related bias should be eliminated.

### 2.5 Connectivity matrix generation

For both atlases (Symmetrical atlas and Sphere atlas), in order to precisely match the parcels to the individual space in which tractography was performed, the original subject-wise T1 image was registered to the MNI T1 template (MNI 152 ICBM 2009a_nlin_hd_1mm) using high dimensional nonlinear registration within the software package ANTs (Avants et al., 2011) and the inverse transform was applied to the parcellation in order to project it into single subject space. Adjacency matrices were constructed by combining the tractograms with the subject's GM parcellation in native space. Streamlines were assigned to the closest node within a 2-mm radius of each streamline endpoint. Each streamline termination was assigned to the nearest grey matter parcel within a 2 mm search radius, which ensures that fiber terminations near the gray-matter boundary, where the diffusion signal becomes noisier and weaker, are adequately captured.

### 2.6 Matrix thresholding

It has been shown that probabilistic algorithms yield inherently noisy connection matrices, at least at the single subject level, and hence likely contain numerous false positives. To reduce false positive rate the matrices were thresholded using a consistency approach (Roberts et al., 2017). More specifically, given a certain number of subjects, a consistency matrix consisting of elements τ_ij_ was calculated. For each edge E (i.e. the adjacency matrix element connecting regions *i* and j), τ(E)=σ(E)/μ(E) was calculated, where the mean μ(E) and standard deviation σ(E) are defined across subjects. The edges with the top 30% consistency values across subjects (i.e. bottom 30% values of τ) were retained in all subjects for group analyses. The consensus connectivity matrix we have obtained is available upon request.

### 2.7 Graph analysis: Modularity and hub computation

Modularity is a global measure of how well a network can be decomposed into a set of sparsely interconnected but densely intraconnected modules, and can be a valuable tool in identifying the functional blocks within a network. In this paper, following prior work (Betzel et al., 2016), network modularity was estimated via the following steps: (i) Modularity was first estimated using the Newman–Girvan algorithm. Each module is extracted as a group of non-overlapping nodes by maximizing the number of within-module links and minimizing the number of between-module links among those nodes. (ii) A Louvain-like algorithm was then used to maximize modularity (1000 iterations). The output of the Louvain algorithm is a set of community assignments, that can be slightly different across iterations. (iii) A consensus partition representing the average community structure was calculated (Lancichinetti and Fortunato, 2012). Once the optimal community structure was defined, within-module degree *z*-scores and participation coefficients were calculated. The within-module degree *z*-score measures how ‘well connected’ node *i* is to other nodes in the module (Guimerà and Amaral, 2005). On the other hand, the participation coefficient compares the number of links of node *i* to nodes in all clusters with its number of links within its own cluster (Guimerà and Amaral, 2005). The participation coefficient of a node is therefore close to one if its links are uniformly distributed among all the modules, and zero if all its links are within its own module.

Network hubs may be defined according to various network criteria (Betzel et al., 2016). Here, hubs were identified according to aggregate ranking across multiple metrics (Betzel et al., 2016). A region's “hubness” was defined jointly based on three indices of centrality: node strength, local efficiency, and betweenness centrality. Nodes with high levels of centrality are thought to facilitate information routing in the network, increasing the overall communication efficiency of a network. A node's strength is the simplest measure of centrality and is defined as the sum of all the edge weights between a node and all the other nodes in the network (Rubinov and Sporns, 2010). Regions with a high nodal strength indicate high connectivity with neighboring nodes. Betweenness centrality of a node is defined as the fraction of all shortest paths in the network that contain a given node. If a node displays a high value of betweenness centrality, it participates in a large number of shortest paths and has an important role in information transfer within a network. Finally, local efficiency reveals how much the system is fault tolerant, by showing how efficient communication is between the first neighbors of a given node when the node is removed (Latora and Marchiori, 2001).

The regions which fell in the top 30% in any of these metrics were defined as hub. “Hubness” values of 3, 2 and 1 were assigned to regions falling in the top 30% for exactly 3, 2 and 1 metrics respectively. As mentioned above, in order to forego possible dependence of structural connectivity estimates on region volume, the Sphere atlas was employed in this analysis.

### 2.8 Multimodal vestibular network

Modularity and hubness analyses were restricted to a large representation of the multimodal vestibular network, including anatomical regions that are reported to respond to vestibular stimulation in humans. This choice was driven by our anatomical parcellation. When the location of vestibular response within an anatomical subdivision was uncertain, we included the whole anatomical region. For example, as vestibular neurons are scattered across the entire thalamus with a potential clustering in the ventrolateral and posterolateral nuclei (Dieterich et al., 2005; Lopez and Blanke, 2011), we included the whole thalamus in our analysis. Similarly, since vestibular stimulation studies in humans revealed activity spread across the hippocampal formation and para-hippocampal gyrus (Bottini et al., 1994; Janzen et al., 2008; Stephan et al., 2005; Suzuki et al., 2001; Vitte et al., 1996) we included this region in its entirety. Also, we included the MT/MST visual motion complex (MT+) plus the adjacent human V5 region (hOc5), insular cortex and opercula, and cingulate cortex including Brodmann area 23c that was described as the vestibular cingulate area CSv (Cardin and Smith, 2010), postcentral gyrus, precuneus, inferior parietal lobule, superior parietal lobule, posterior superior temporal sulcus, inferior temporal gyrus, middle temporal gyrus, precentral gyrus, inferior frontal gyrus, for a total of 172 regions (Table 1, in bold). Our analysis focused on the cerebral cortex and thalamus. Future studies will focus on the vestibular cerebellum, that is connected to the cortex through fastigial and vestibular nuclei (Kirsch et al., 2016). This choice is motivated by the higher resolution (affordable mostly with ultra-high filed imaging) necessary to characterize small structures near the deep gray matter (nuclei, brainstem) (Jeurissen et al., 2019).

**Table 1:** List of abbreviations. Areas in bold are those selected as belonging to the multimodal vestibular network.

Location	Label	Area	Atlas
Superior frontal gyrus	8dl	dorsolateral area 8	Fan
	8m	medial area 8	Fan
	9l	lateral area 9	Fan
	9m	medial area 9	Fan
	6dl	dorsolateral area 6	Fan
	6m	medial area 6	Fan
	10l	lateral area10	Fan
Middle frontal gyrus	IFJ	inferior frontal junction	Fan
	IFS	inferior frontal sulcus	Fan
	8vl	ventrolateral area 8	Fan
	6vl	ventrolateral area 6	Fan
	9/46d	dorsal area 9/46	Fan
	9/46v	ventral area 9/46	Fan
	10m	medial area 10	Fan
	**44**	**area 44**	Eickhoff
Inferior Frontal gyrus	**44d**	**dorsal area 44**	Fan
	**44op**	**opercular area 44**	Fan
	**44v**	**ventral area 44**	Fan
	**45**	**rostral area 45**	Eickhoff
	**45c**	**caudal area 45**	Fan
Orbital gyrus	Fp1	Frontpolar 1	Eickhoff
	11l	lateral area 11	Fan
	12/47o	orbital area 12/47	Fan
	14m	medial area 14	Fan
Precentral gyrus	4hf	area 4 (head and face region)	Fan
	4ll	area 4 (lower limb region)	Fan
	4t	area 4 (trunk region)	Fan
	**4tl**	**area 4 (tongue and larynx region)**	Fan
	4ul	area 4 (upper limb region)	Fan
	**6cdl**	**caudal dorsolateral area 6**	Fan
	**6cvl**	**caudal ventrolateral area 6**	Fan
Superior temporal gyrus	41/42	area 41/42	Fan
	TE1.0 1.2	auditory TE1.0 and TE1.2	Fan
	22r	rostral area 22	Fan
	38l	lateral area 38	Fan
Middle temporal gyrus	**V5/MT+**	**visual motion complex**	Fan
	**37dl**	**dorsolateral area37**	Fan
	**aSTS**	**anterior superior temporal sulcus**	Fan
	21c	caudal area 21	Fan
	21r	rostral area 21	Fan
Inferior temporal gyrus	37elv	extreme lateroventral area37	Fan
	37vl	ventrolateral area 37	Fan
	20iv	intermediate ventral area 20	Fan
	20il	intermediate lateral area 20	Fan
	20cl	caudolateral area 20	Fan
	20cv	ventrolateral area 20	Fan
	20r	rostral area 20	Fan
Fusiform Gyrus	FG2	Fusiform Gyrus 2	Eickhoff
	FG4	Fusiform Gyrus 4	Eickhoff
	37lv	lateroventral area37	Fan
	37mv	medioventral area37	Fan
Parahippocampal gyrus	**EC**	**Entorhinal Cortex**	Eickhoff
	**35/36c**	**caudal area 35/36**	Fan
	**28/34**	**area 28/34**	Fan
	**TL**	**area TL (lateral posterior parahippocampal gyrus)**	Fan
	**TH**	**Area hippocampotemporalis**	Fan
Hippocampus proper	**CA1**	**Cornu Ammonis 1**	Eickhoff
	**CA2**	**Cornu Ammonis 2**	Eickhoff
	**CA3**	**Cornu Ammonis 3**	Eickhoff
	**DG**	**dentate gyrus**	Eickhoff
	**Subiculum**		Eickhoff
	**cHipp**	**caudal hippocampus**	Fan
	**rHipp**	**rostral hippocampus**	Fan
Insula	**Id1**	**dysgranular insula**	Eickhoff
	**Ig1**	**granular insula 1**	Eickhoff
	**Ig2**	**granular insula 2**	Eickhoff
	**G**	**Hypergranular insula**	Fan
	**dIa**	**dorsal agranular insula**	Fan
	**dId**	**dorsal dysgranular insula**	Fan
	**dIg**	**dorsal granular insula**	Fan
	**vIa**	**ventral agranular insula**	Fan
	**vId/vIg**	**ventral dysgranular and granular insula**	Fan
	**TI**	**area TI(temporal agranular insular cortex)**	Fan
Parietal operculum	**OP1**	**secondary somatosensory area (SII)**	Eickhoff
	**OP2**	**parieto insular vestibular cortex (PIVC)**	Eickhoff
	**OP3**	**ventral somatosensory area (VS)**	Eickhoff
	**OP4**	**parietal ventral area (PV)**	Eickhoff
Postcentral gyrus	3a	area 3a	Eickhoff
	1	area 1	Eickhoff
	1/2/3ll	area1/2/3 (lower limb region)	Fan
	1/2/3ulhf	area 1/2/3(upper limb, head and face region)	Fan
Cingulate gyrus	**23c**	**caudal area 23**	Fan
	**23d**	**dorsal area 23**	Fan
	**23v**	**ventral area 23**	Fan
	**24cd**	**caudodorsal area 24**	Fan
	**24rv**	**rostroventral area 24**	Fan
	**32p**	**pregenual area 32**	Fan
	**32sg**	**subgenual area 32**	Fan
Inferior parietal cortex	**PF**	**Area supramarginalis**	Eickhoff
	**PFcm**	**Area supramarginalis columnata magnocellularis (posterior)**	Eickhoff
	**PFm**	**Area supramarginalis magnocellularis**	Eickhoff
	**Pfop**	**Area supramarginalis opercularis**	Eickhoff
	**PFt**	**Area supramarginalis tenuicorticalis**	Eickhoff
	**Pga**	**Area angularis, anterior**	Eickhoff
	**PGp**	**Area angularis, posterior**	Eickhoff
	**40c**	**caudal area 40**	Fan
	**40rd**	**rostrodorsal area 40**	Fan
	**PIC**	**rostroventral area 40**	Fan
	**39c**	**caudal area 39**	Fan
	**39rd**	**rostrodorsal area 39**	Fan
	**39rv**	**rostroventral area 39**	Fan
Intraparietal sulcus	**hIP1**	**human intraparietal 1**	Eickhoff
	**hIP2**	**human intraparietal 2**	Eickhoff
	**hIP3**	**human intraparietal 3**	Eickhoff
Superior parietal lobe	**5Ci**		Eickhoff
	**5L**		Eickhoff
	**5l**	**lateral area 5**	Eickhoff
	**7A**		Eickhoff
	**7P**		Eickhoff
	**7c**	**caudal area 7**	Fan
	**7ip**	**intraparietal area 7**	Fan
	**7PC**	**postcentral area 7**	Fan
	**7r**	**rostral area 7**	Fan
Precuneus	**PEp**	**Parvicellular superior parietal area**	Eickhoff
	**5M**		Eickhoff
	**7M**		Eickhoff
	**5m**	**medial area 5**	Fan
	**7m**	**medial area 7**	Fan
	**dmPOS**	**dorsomedial parietooccipital sulcus**	Fan
	**31**	**area 31**	Fan
Occipital cortex	hOC3v	human ventral V3	Eickhoff
	hOC4v	human ventral V4	Eickhoff
	hOC4la	human lateral anterior V4	Eickhoff
	**hOC5**	**human V5**	Eickhoff
	rLinG	rostral lingual gyrus	Fan
Thalamus	**Thal Motor**		Eickhoff
	**Thal Parietal**		Eickhoff
	**Thal Prefrontal**		Eickhoff
	**Thal Premotor**		Eickhoff
	**Thal Somatosensory**		Eickhoff
	**Thal Temporal**		Eickhoff
	**Thal Visual**		Eickhoff
Basal forebrain	BF		Eickhoff

### 2.9 Structural connectivity lateralization

To study the lateralization of a region's overall connectivity we used the Symmetrical atlas. We defined a regional structural connectivity lateralization index L as the median of a vector **L** whose components were the normalized difference between the right and left connectivity thresholded matrix elements (RightW_ni_ - LeftW_ni_) defining connections of a given region n with all the other regions *i* (with *i* ranging from 1 to N=222 regions in each hemisphere -1).

$$\begin{array}{ccc} L & = & \left[\ldots \ldots ,\left(\text{Right}W_{\text{ni}}-\text{Left}W_{\text{ni}}\right)/\left(\text{Right}W_{\text{ni}}+\text{Left}W_{\text{ni}}\right),\ldots \ldots \right];\\ i & = & \left[1,\ldots ..N-1\right]; \end{array}$$

The normalization uses the sum of right and left connectivity matrix elements so as to vary between -1 (area fully left lateralized in connections with most regions) and 1 (area fully right lateralized in connections with most regions). Thus, by considering the median of the distribution, this statistics would reflect the number of stronger connections of a given area on one side of the brain compared to the contralateral homologue area. Note, that a given value of L could result not only from stronger connections of the area of one side of the brain compared to the contralateral one, but it would be also influenced by a potential higher number of suprathreshold connections made by one of the two areas. We first tested the null hypothesis of no lateralization (i.e. L = 0, where L is the median of **L**) in right handed individuals only (n = 794 participants) using a signed rank Wilcoxon test across the elements of **L**, followed by Bonferroni correction across the length of **L** (p_corr_ <0.05). We then extended the analysis to the whole sample of participants to study the effect of handedness and gender on lateralization. We studied the effect of handedness through correlation of each participant's regional L index with handedness scores (Spearman correlation, p_corr_ <0.05), and gender effects through comparison of female and male regional L index (rank sum Wilcoxon test, p_corr_ <0.05).

### 2.10 Comparison between connectivity of OP2 and PIC

To compare the connectivity of OP2 and PIC we used the Sphere atlas on individual connectivity matrices. We studied regional differences (RD) by comparing individual elements of the connectivity matrix across subjects as follows:

$$\begin{array}{c} \text{Individual}\;\text{RD}=\left(W_{i}\left(\text{PIC}\right)-W_{i}\left(\text{OP}2\right)\right)\\ \left(\text{with}\;i\;\text{indexing}\;\text{all}\;\text{regions}\right) \end{array}$$

In order to forego assumptions about distribution shape, this analysis was performed through a bootstrap approach. In particular, for every region, starting from RD values across subjects, random sign flipping within each subject followed by averaging was performed 10^5^ times in order to build an empirical distribution of the null hypothesis (i.e. no connectivity differences of that particular region with PIC vs OP2). The real difference was then tested against this distribution by examining the quantile in which it fell. The resulting p-values were subjected to Bonferroni correction across regions and a value of p_corr_ <0.05 was considered statistically significant.

## 3 Results

### 3.1 Modularity

Modularity was calculated from the Symmetrical atlas in the multimodal vestibular network (Table 1 in bold) in the 794 right handers. The right and left hemispheres included 3 and 4 modules respectively, indicating that the vestibular network is more integrated in the right hemisphere. Here, regions were grouped into a parietal-insular-prefrontal module (Fig. 1, light green), into a visual module (Fig. 1, purple), and into a limbic-subcortical module (Fig. 1, yellow). The parietal-insular-prefrontal module comprised the superior parietal cortex, including the ventral intraparietal (VIP) region (7ip, see (Glasser et al., 2016)), the intra-parietal sulcus (hIP1/2/3) and supramarginal gyrus, the temporo-parietal-occipital junction and the posterior superior temporal sulcus (rpSTs, cpSTs), the insula, parietal and frontal opercula and frontal regions (premotor cortex and IFg) (Fig. 1 in light green). In the left hemisphere, this module was more restricted compared to the right hemisphere, since it did not include the superior parietal cortex and the more dorsal division of the supramarginal gyrus (Fig. 1 in light red). These latter parietal areas, instead, clustered with the dorsal visual stream (Fig. 1 in dark green), which, on the other hand, formed an isolated cluster on the right hemisphere (Fig. 1 in purple).

In the right hemisphere, the limbic-subcortical module extended medially to the hippocampal formation, cingulate cortex, parieto-occipital regions, thalamus, and laterally to dorsolateral caudal region of BA 6 (6cdl), corresponding to supplementary eye fields (SEFs) (Grosbras et al., 1999), and ventral insula (Fig. 1 in yellow). On the left side this module was split in two, one comprising the hippocampal formation and 6cdl (Fig. 1 in blue), the other the remaining cingulate and parieto-occipital regions (Fig. 1 in dark red).

### 3.2 Hubness

**Fig. 2:**
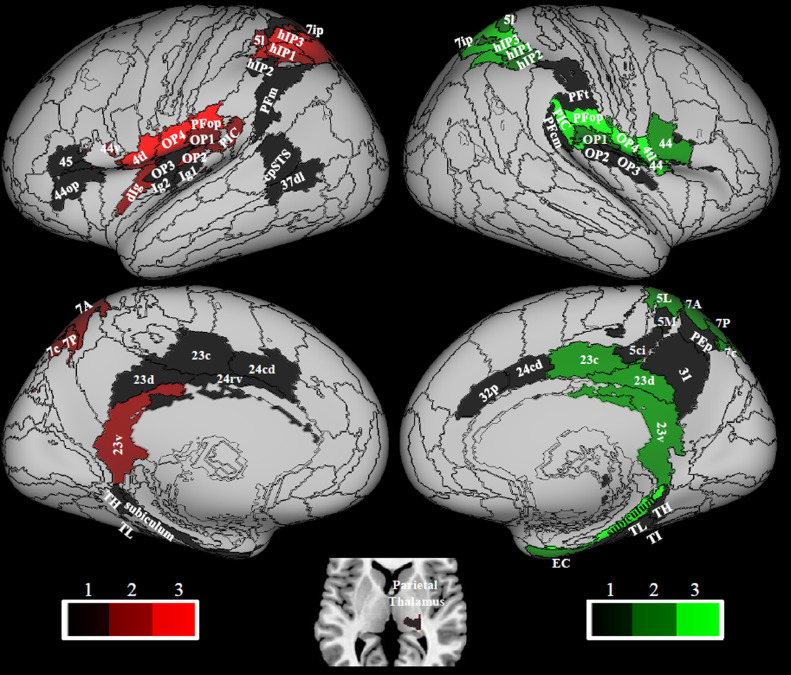
Hubness. Areas that show high hubness, i.e. the higher scores in 3, 2 or 1 metrics (nodal strength, betweenness centrality and local efficiency); 30% of areas within the vestibular network with higher values of each metric were selected for ranking. Data from 794 right handed individuals. The sphere atlas was used to calculate these metrics (see 2.4.). Regions in the left hemisphere are colored in red while in the right hemisphere are colored in green. Regions from the composite atlas are overlapped onto the Conte69 inflated brain in workbench viewer (Glasser et al 2016).

Hubness was calculated from the Sphere atlas in the multimodal vestibular network (Table 1 in bold), by using only data from the 794 right handers. Regions with the highest hubness scores were concentrated in the posterior peri-sylvian regions (right PIC, bilateral PFop, OP4) bilateral 4tl and intraparietal sulcus (bilateral hIP1 and hIP3, right 5l, left 7ip) plus the right subiculum (Fig. 2, Table 3). Regions with medium hubness score included the parietal opercula (left OP2, bilateral OP1) and left PIC, right intraparietal cortex hIP2, the left anterior insula (dIg), bilateral regions in area 44, right enthorinal cortex and bilateral posterior cingulate regions (bilateral 23v, right 23d and 23c) (Fig. 2, Table 3).

The posterior insula (Ig1, Ig2) and ventral stream visual areas (cpSTS, 37dl) showed low hubness on the left, while parietal thalamus showed low hubness on the right (Fig. 2, Table 3).

### 3.3 Hemispheric structural lateralization

**Fig. 3:**
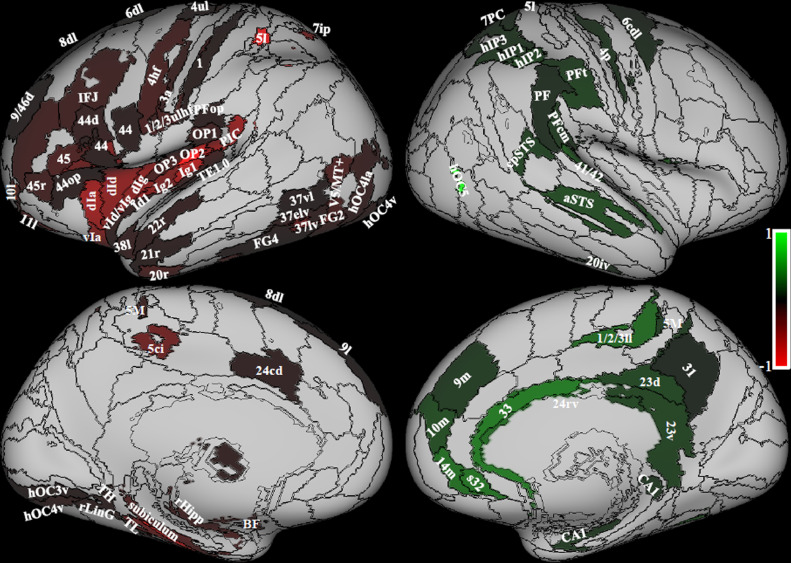
Structural lateralization. On the left in red, areas that show a significant left laterality index; on the right in green areas that show a significant right laterality index; (Wilcoxon signed test, p < 0.05 Bonferroni corrected). Data from 794 right handed individuals. The color bars represent the range of laterality index. Regions from the composite atlas are overlapped onto the Conte69 inflated brain in workbench viewer (Glasser et al 2016).

The whole brain analysis performed with the Symmetrical atlas on right handed subjects, showed several regions with significant lateralization of overall structural connectivity (Wilcoxon signed rank test, Bonferroni corrected for number of areas at p <0.05, Fig. 3, Table 2). In general, more regions showed structural connectivity lateralization to the left side. In agreement with the literature, Brodmann areas 44 and 45, that include the left lateralized Broca's area for speech production and regions in the primary motor area, were left lateralized. Also, the insula, the parietal and frontal opercula (OP1/2/3, PFop, 44op), the rostro-ventral portion of the supramarginal gyrus (PIC) and areas in the superior parietal lobe were lateralized to the left side. In addition, prefrontal regions (IFJ, 6, 8, 10, 11, 46), visual regions in the ventral stream (including the visual word form area), inferior and middle temporal pole, and regions of the hippocampal formation showed left side predominance. Right lateralized regions were located in the perisylvian posterior region of the supramarginal gyrus (PFcm, PF, PFt), area 41/42 and superior temporal sulcus, vast portions of cingulate gyrus and precuneus and in CA1 in the hippocampus.

**Table 2:** Structural connectivity Lateralization (L) of areas to the left or right brain hemisphere. Lateralization length is the total number of above threshold connections of each area in both hemispheres.

Area	Left L	L length	pBonf	Area	Right L	L length	pBonf
7pc	-1.00	65	0.00	hOc5	1.00	28	0.02
28/34	-1.00	49	0.00	37mv	0.43	45	0.00
40c	-1.00	75	0.00	33	0.39	207	0.00
35/36c	-1.00	36	0.00	s32	0.35	70	0.00
OP2	-1.00	54	0.00	14m	0.34	81	0.00
5l	-1.00	74	0.00	TE1.0/TE1.2	0.33	162	0.00
Ig1	-0.63	149	0.00	24rv	0.31	205	0.00
PIC	-0.54	139	0.00	1/2/3ll	0.31	166	0.00
dIa	-0.54	174	0.00	41/42	0.29	211	0.00
7ip	-0.51	99	0.00	hIP2	0.24	151	0.00
vIa	-0.50	159	0.00	10m	0.20	153	0.00
G	-0.49	123	0.00	aSTS	0.19	226	0.00
dId	-0.46	211	0.00	hIP1	0.18	151	0.00
dIg	-0.46	149	0.00	A23d	0.17	310	0.00
11l	-0.44	32	0.00	23v	0.17	326	0.00
44v	-0.43	135	0.00	PFt	0.16	204	0.00
TL	-0.41	63	0.00	cpSTS	0.16	165	0.00
37lv	-0.39	80	0.00	PFcm	0.13	182	0.00
Ig2	-0.39	213	0.00	CA1	0.13	143	0.00
45c	-0.35	122	0.00	9m	0.13	238	0.00
5Ci	-0.33	113	0.00	20iv	0.13	119	0.00
20r	-0.27	130	0.00	5m	0.13	230	0.00
rHipp	-0.24	296	0.00	hIP3	0.13	225	0.00
Subiculum	-0.23	221	0.00	4p	0.12	152	0.00
V5MT+	-0.21	132	0.00	Lobule IX (Hem)	0.12	119	0.00
45	-0.20	238	0.00	7PC	0.10	192	0.00
10l	-0.20	103	0.00	6cdl	0.09	242	0.00
4hf	-0.20	220	0.00	PF	0.08	267	0.00
1/2/3ulhf	-0.18	145	0.00	Lobule VIIa crusI (Hem)	0.07	190	0.00
TE 10	-0.18	166	0.00	Lobule VIIa crusII (Hem)	0.07	178	0.00
946v	-0.18	220	0.00	dlPu (dorsolateral putamen)	0.07	353	0.00
7r	-0.18	87	0.00	31	0.06	283	0.02
OP1	-0.18	149	0.00				
OP3	-0.17	127	0.00				
45r	-0.17	160	0.00				
BF (Ch 4)	-0.17	138	0.00				
FG2	-0.16	108	0.00				
IFJ	-0.16	226	0.00				
mOccG	-0.15	100	0.00				
38l	-0.15	156	0.00				
TE 11	-0.15	167	0.00				
Ventral Dentate Nucleus	-0.15	62	0.00				
hOc4v	-0.14	149	0.00				
3a	-0.14	73	0.00				
hOc4la	-0.14	194	0.00				
Lobule X (Hem)	-0.13	83	0.00				
44d	-0.12	172	0.00				
vId/vIg	-0.11	249	0.00				
20rv	-0.11	171	0.00				
Lobule V (Hem)	-0.11	123	0.00				
22r	-0.11	162	0.00				
rLinG	-0.10	129	0.00				
37elv	-0.10	115	0.02				
Thal Temporal	-0.10	354	0.00				
24cd	-0.09	116	0.00				
Thal Prefrontal	-0.09	329	0.00				
Lobule I IV (Hem)	-0.09	266	0.00				
44op	-0.09	179	0.00				
Pfop	-0.08	150	0.00				
hOc3v	-0.08	170	0.00				
44	-0.08	237	0.00				
21r	-0.08	180	0.02				
4ul	-0.08	200	0.00				
FG4	-0.08	128	0.00				
1	-0.07	210	0.04				
9/46d	-0.07	219	0.02				
6dl	-0.07	284	0.00				
Thal Parietal	-0.07	356	0.00				
9l	-0.06	258	0.00				
37vl	-0.06	167	0.00				
8dl	-0.04	258	0.04				
vmPu	-0.03	288	0.04

**Table 3:** Hubness. L=Left, R=Right.

Hubness =3	Hubness=2		Hubness=1		
4tl L/R	44v L	23d R	44v R	cpSTS L	OP3 L/R
5l R	5l L	23v L/R	44op L	20rv L/R	PFcm R
7ip L	7ip R	23c R	45c L/R	7r L	7PC L
PIC R	PIC L	OP1 L/R	37dl L	7m R	Thal Parietal R
PFop L/R	7r R	OP2 L	OP2 R	31 R	5Ci R
hIP1 L/R	7c L/R	5L R	35/36r L/R	23d L	Ig2 L
hIP3 L/R	1/2/3tonIa L/R	7A L/R	35/36c R	24rv L	hIP2 L
OP4 L/R	dIg L	Entorhinal Cortex R	TL L	32p R	Entorhinal Cortex L
Subiculum R	7PC R		Subiculum L	24cd L/R	Ig1L
	7P L/R		28/34 L/R	23c L	PFt R
	hIP2 R		TI L/R	PFm L	5M R
	44 R		TH L/R	45 L	

By extending the analysis to the whole sample of participants (n=974) independently of handedness we found that structural connectivity lateralization did not depend on handedness (Spearman correlation between handedness and individual L, all p's > 0.001, not surviving Bonferroni correction for the number of regions). Further, structural connectivity lateralization did not depend on gender, either (Wilcoxon ranksum test, all p's > 0.005, not surviving Bonferroni correction across regions).

### 3.4 Comparison between structural connectivity of OP2 and PIC

#### 3.4.1 PIC structural connectivity

**Fig. 4:**
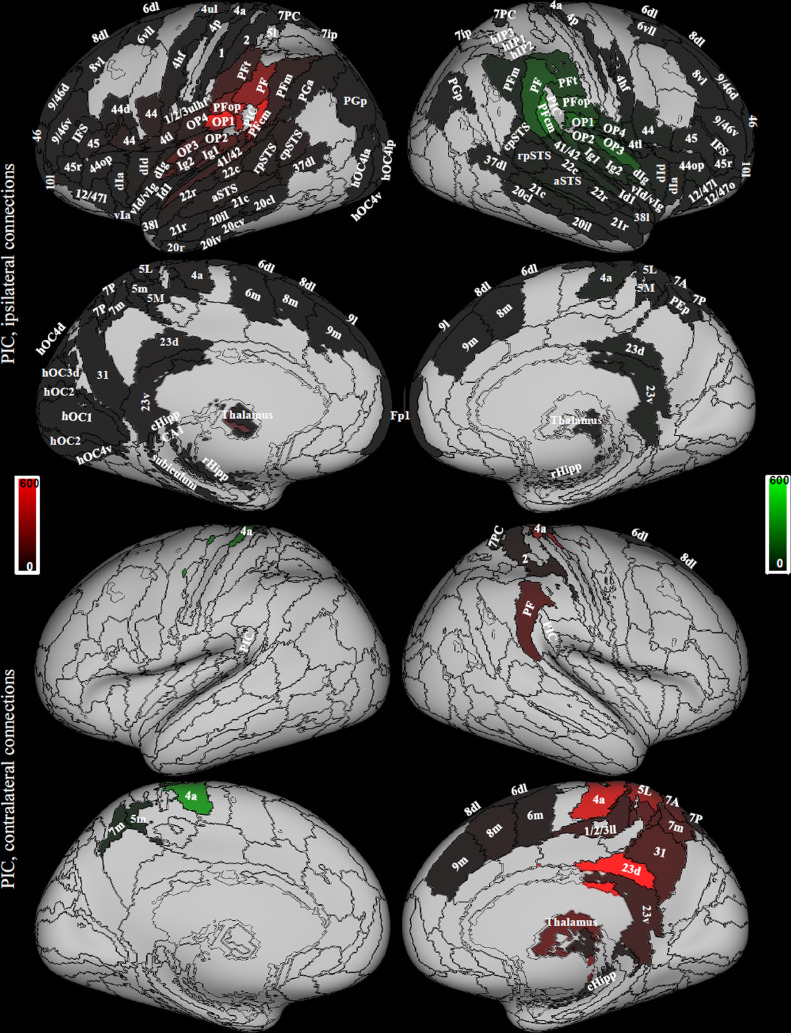
Areas connected with PIC. Data from 794 right handed individuals, Symmetrical atlas (see 2.4). The red color indicates areas connected to the left PIC, the green color areas connected to the right PIC. The scale refers to streamline count. Regions from the composite atlas are overlapped onto the Conte69 inflated brain in workbench viewer (Glasser et al 2016).

The region defined as PIC in our atlases is located posterior to OP1, OP2 and PFop, and anterior to PFcm (x=-46, y=-33, z=24, left; x=51, y=-27, z=28 right; MNI coordinates of the PIC centroid). It comprises the anterior portion of the rostroventral Brodmann area 40 defined in the Fan atlas. The name PIC (posterior insular cortex) is kept for historical reasons, though the region is not in the insula (Beer et al., 2009; Frank et al., 2016; Frank and Greenlee, 2018; Sunaert et al., 1999). We found that PIC is a hub of the selected multimodal vestibular network and shows left lateralized structural connectivity. It is connected bilaterally to the medial superior parietal regions including VIP (7r, 7ip) and to the majority of the thalamus, and ipsilaterally to the insula, perisylvian regions, frontal premotor regions, several occipital and temporal areas, the posterior cingulate cortex and the rostral hippocampus (Fig. 4).

#### 3.4.2 OP2 structural connectivity

**Fig. 5:**
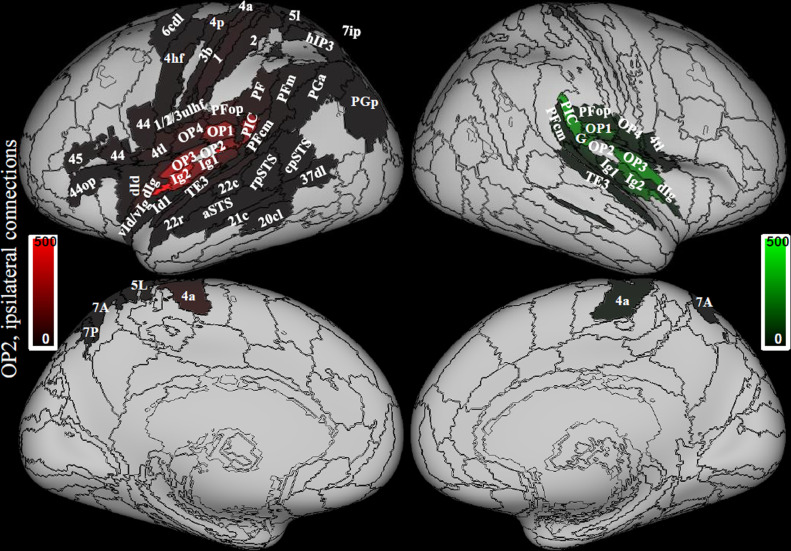
Areas connected with OP2 are only ipsilateral. Data from 794 right handed individuals, Symmetrical atlas (see 2.4). The red color indicates areas connected to the left OP2, the green color areas connected to the right OP2. The scale refers to streamline count. Regions from the composite atlas are overlapped onto the Conte69 inflated brain in workbench viewer (Glasser et al 2016).

The region we defined as OP2, corresponds to the homonym region in the Eickhoff atlas (x=-36, y=-25, z=18, left; x=36, y=-22, z=17, right; MNI coordinates of OP2 centroid). In the literature, it has been considered functionally as PIVC (parieto-insular vestibular cortex) (zu Eulenburg et al., 2012). We also found that OP2 is a hub of the multimodal vestibular network and shows left lateralized structural connectivity. It is connected ipsilaterally to the rest of the insula and perisylvian regions, the superior parietal cortex including VIP (A7r, A7ip) and the somatosensory cortex (Fig. 5).

**Fig. 6:**
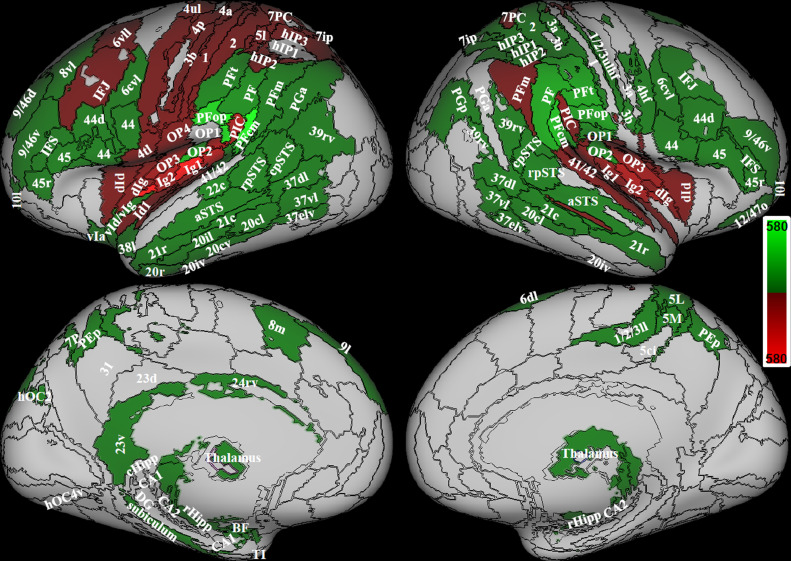
Comparison between PIC and OP2 connectivity. Areas that show higher connectivity to PIC than OP2 and viceversa (green: PIC > OP2, red: OP2 > PIC). For simplicity, only comparisons between ipsilateral connections are shown. Data from 794 right handed individuals. The sphere atlas was used to calculate connectivity strength (see 2.4). Regions from the composite atlas are overlapped onto the Conte69 inflated brain in workbench viewer (Glasser et al 2016).

Direct comparison of OP2 and PIC structural connectivities showed that overall, PIC is more connected to the visual ventral stream, the superior temporal sulcus, superior, middle and inferior temporal gyrus, the supramarginal and angular gyrus, inferior and middle frontal gyrus, and the thalamus (Fig. 6 green, Supplementary table 1) as compared to OP2. In the right hemisphere, PIC is more connected to the superior parietal lobe (SPL), the intraparietal sulcus, somatosensory, motor and premotor areas than OP2. Finally, PIC is more connected than OP2 also to medial regions of the brain, such as the cingulate posterior region and the hippocampal formation. However, OP2 is more connected than PIC to adjacent regions in the parietal operculum and to the insula, and left OP2 is more connected than PIC to SPL, the intraparietal sulcus, somatosensory, motor and premotor areas (ranksum Wilcoxon test, p_corr_ <0.05, corrected for the number of regions).

## 4 Discussion

We determined the topological features of the structural connectivity patterns in the vestibular cortex and the lateralization of its anatomical connections in a large and high-quality multi-shell diffusion dataset (*n=974*) with high spatial resolution. To this end, we employed state-of-the-art tractography methods and carefully selected and crafted parcellations of atlas of the human cerebral cortex.

We found that the vestibular network displays higher integration in the right hemisphere (relative to the left), as denoted by the lower number of modules. In the right hemisphere, three modules were identified: a parietal-insular-prefrontal set of regions, a visual module, and a limbic-subcortical group of areas. This latter group included the hippocampal formation, the cingulate cortex, and the thalamus. In the left hemisphere, the parietal-insular-prefrontal module was split into a sub-module comprising insula, opercula and prefrontal regions, and another sub-module including the supramarginal gyrus and intraparietal cortex. The higher level of integration (lower number of modules) in the right hemisphere may reflect a right lateralization of visuo-spatial processes such as spatial navigation and of the processing of vestibular inputs (Dieterich et al., 2003; Jager and Postma, 2003; Kirsch et al., 2018, 2016; Maguire et al., 1998).

We also found that the multimodal vestibular network shows right structural connectivity lateralization in the most posterior areas of the supramarginal gyrus (PFcm, PF, PFt), in the intraparietal cortex and STS, and, in contrast, left lateralization in the insula, parietal and frontal opercula and inferior frontal cortex.

### 4.1 OP2 and PIC

We focused our analysis on OP2 and PIC, two regions that have been indicated as human homologues of PIVC and VPS in monkeys, respectively (Frank et al., 2016, 2014; Frank and Greenlee, 2018; zu Eulenburg et al., 2012). Both PIVC in monkey and OP2 in humans respond to vestibular inputs but not visual stimuli (Chen et al., 2010; zu Eulenburg et al., 2012), while VPS in monkeys and PIC in humans respond to both vestibular and visual stimuli (Chen et al., 2011b; Frank et al., 2016). As the VPS and PIVC are ‘hub’ vestibular regions in monkeys (Guldin et al., 1992), we expected that OP2 and PIC were ‘hub’ vestibular regions in humans (Frank et al., 2016). Consistently with our predictions, PIC showed the highest degree of hubness in the right hemisphere while both PIC and OP2 showed a medium degree of hubness in the left hemisphere. It is important to note that the hubness of a particular region does not reflect the local strength of that region alone, but also represents the connectivity features of the neighboring regions (as assessed by the local efficiency and betweenness centrality) (Rubinov and Sporns, 2010). For example, when a region has high local efficiency, there is also a high likelihood that its neighboring regions are interconnected with each other. This means that an area with a left-lateralized structural connectivity pattern (as defined by high local strength) can also have a higher degree of ‘hubness’ in the opposite hemisphere (right rather than left). We found that this was the case for PIC, which showed structural connectivity lateralized to the left hemisphere, but higher ‘hubness’ on the right. Conversely, the structural connectivity of OP2 was lateralized to the left hemisphere, where it also showed higher ‘hubness’ relative to the right hemisphere.

In addition, the areas connected to PIC were more diffuse and bilateral relative to the areas connected to OP2. PIC was also more connected to visual areas, the posterior cingulate region, retro-splenial cortex, hippocampus, and inferior parietal cortex, a set of regions consistently implicated in visuo-spatial navigation. On the other hand, the right OP2 showed, overall, a more limited pattern of connectivity which was particularly related to the insula. In addition, the connectivity pattern of the left OP2 included somatosensory, motor and premotor regions in the ipsilateral hemisphere. Interestingly, the structural connectivity patterns that we found for PIC and OP2 show strong analogies with those reported by a neuroanatomical study in squirrel monkeys for VPS (area T3) and PIVC, respectively (Guldin et al., 1992). In particular, VPS displays strong connections with parieto-occipital and parieto-temporal regions (area 19), the upper bank of the temporal sulcus (STS-area), anterior cingulate gyrus, and parts of the posterior parietal area 7. In contrast, PIVC is connected with Brodmann's areas 8a, 6, 3a, 3aV, 2, and posterior parietal area 7ant (Guldin et al., 1992) .

Overall, these results suggest that PIC and OP2 are involved in processing distant space information and peri-personal space, respectively (Ventre-Dominey, 2014). These findings are also in keeping with a previous study in which PIVC and PIC were localized in 15 individual brains through fMRI (Wirth et al., 2018). PIC and PIVC were identified through caloric vestibular stimulation, which can activate both areas, and visual motion stimulation, that is known to activate PIC but not PIVC (Wirth et al., 2018). Wirth et al. found that the region identified as PIVC in the group average overlapped with OP2 and OP3, while PIC overlapped with PIC in our atlas and the anterior ventral sections of PFcm and PF. In Wirth et al. study, PIVC showed significantly more structural connectivity (relative to PIC) with the anterior insula and Heschl's gyrus (area 41/42) in both hemispheres, and significantly less structural connectivity (as compared to PIC) in the supramarginal gyrus and superior temporal sulcus (Wirth et al., 2018). Despite the fact that PIVC and PIC showed inter-individual variability when localized through functional activation (Wirth et al., 2018), thus spreading across cytoarchitectonic regions, structural connectivity patterns are in good agreement between Wirth et al. study and our study.

Based on these differences in the extent of putative areas, we feel that some uncertainty should be allowed in the analogy between PIVC and VPS in the monkey and OP2 and PIC in humans by considering the possibility that they overlap with adjacent regions as OP3 and the anterior ventral sections of PFcm and PF, respectively. In this context, it is interesting to note that OP3 shows the same left structural connectivity lateralization as OP2, while PFcm and PF, contrary to PIC, shows right lateralization.

### 4.2 Lateralization

Previous studies suggested that the vestibular function is lateralized to the right hemisphere in right-handed individuals and to the left hemisphere in left-handed people (Dieterich et al., 2003; Janzen et al., 2008; Kirsch et al., 2018; Lopez et al., 2012). Using caloric vestibular stimulation, the higher vestibular response in the right hemisphere in right handers has been found in the postcentral gyrus, superior and inferior parietal lobe, anterior cingulum, frontal gyrus (Dieterich et al., 2003), and in the superior temporal gyrus and insular gyrus V when using sound evoked vestibular stimulation (Schlindwein et al., 2008), but neither in PIVC nor PIC. Even though lateralization in our study concerns structural connectivity and can therefore not be directly compared to functional activation lateralization, we also found right lateralization of the majority of these regions, in particular in the superior parietal lobe (7PC, 5l), in regions surrounding the intraparietal sulcus (hIP1/2/3), in the supramarginal gyrus (PFcm, PF, PFt), in the cingulate gyrus (BA 33) and in the superior temporal gyrus (cpSTS, aSTS) (Fig. 3, green).

A meta-analysis of caloric, galvanic and sound evoked vestibular stimulation imaging studies, showed that vestibular stimulation involves a larger volume of activation in the right hemisphere during stimulation of the right ear than in the left hemisphere during stimulation of the left ear across parietal, temporal and insular cortices (Lopez et al., 2012). However, due to the low spatial resolution inherent to the meta-analysis technique, this study considered the functional activity of each hemisphere as a whole, thus not allowing to draw conclusions about differences within the posterior peri-sylvian cortex.

Our results are in agreement with a study that performed a comprehensive taxonomy of functional lateralization in the brain distributed along four functional axes: symbolic communication, perception/action, emotion, and decision-making (Karolis et al., 2019). This study reported left functional lateralization of regions corresponding to OP2 and PIC, while right functional lateralization of adjacent posterior peri-sylvian areas in the supramarginal and temporal gyrus for the perception/action function (Karolis et al., 2019).

Finally, a functional connectivity study described the multimodal vestibular cortex as an external circle of symmetric (not lateralized), well-connected multisensory areas (hubs in the superior temporal gyrus, temporo-parietal intersection) organized around an internal circle of asymmetric (lateralized to the right for right-handers and to the left for left-handers) and functionally more specialized core regions in the middle posterior and inferior insula (Kirsch et al., 2018). Though our results are not in accordance with this description, we also found that the ‘external circle’ regions (opercula and supramarginal gyrus) show, in general, higher degrees of hubness as compared to the ‘internal circle’ (insula).

### 4.3 Handedness and lateralization of the vestibular system

Regarding the relationship between handedness and the vestibular system, previous studies have reported that the right hemisphere is dominant for vestibular functions in right-handed individuals while the opposite is true for left-handed people (Dieterich et al., 2003; Kirsch et al., 2018). Our data are not consistent with these previous findings, as we did not find any significant relationship between structural connectivity and handedness. One reason for the inconsistencies between the studies could be represented by the methodological differences between the studies and by the fact that the hemispheric dominance in terms of structural connectivity may not necessarily correspond to functional hemispheric dominance.

With this caveat in mind, it should be nonetheless noted that the lateralization of one function is not necessarily associated with a clear relationship between the hemispheric dominance itself and handedness (Capozzoli, 1999; Scharoun and Bryden, 2014). In other words, although language skills are typically lateralized to the left hemisphere in right-handed people, there is still a large proportion (i.e., 60–70%) of left-handed individuals that retain a left-hemisphere dominance for language or at least a reduced functional asymmetry, rather than a clear right lateralization of the language skills (Capozzoli, 1999; Scharoun and Bryden, 2014). Analogously, an fMRI study of sound-evoked vestibular response in left-handers suggested that processing was bilateral, with only a mild tendency toward the left hemisphere (Janzen et al., 2008). Also, recovery from a left-hemispheric stroke, seems more rapid and complete in left handers than in right-handers with right hemispheric stroke, thus suggesting higher bilateral processing of vestibular stimuli in left-handers than right-handers (Dronkers and Knight, 1989; Vanderploeg, 1986).

### 4.4 Clinical implications

Improving our knowledge of the lateralization of vestibular function may inform clinical understanding of the cortical mechanisms of spatial hemi-neglect and the ‘pusher’ syndrome, two neurological disorders that have been related to ‘cognitive’ aspects of the vestibular function (Brandt et al., 2014; Brandt and Dieterich, 2015). Intriguingly, both disorders are prevalent in patients with right hemisphere damage (Abe et al., 2012; Karnath and Dieterich, 2006; Karnath and Rorden, 2012).

The lateralization of a function also implies that acute lesions in regions in the dominant hemisphere may result in the rapid emergence of symptoms, like those described by patients with acute aphasia or neglect due to a stroke. Our results thus suggest that, in patients with e.g. selective damage to certain cortical vestibular regions, it should in theory be possible to distinguish between left- and right-hemisphere dominance for certain vestibular functions (with the caveat that our findings should be confirmed in functional imaging studies and more importantly in clinical populations). Some studies have already begun to provide support in this direction, for example in the clinical syndrome of subjective visual vertical (SVV) tilt, which has been found to depend on damage in the left insular cortex or to right-sided lesions of the superior temporal gyrus, temporo-parietal junction (TPJ), and dorsal parietal cortex (Baier et al., 2012b; Rousseaux et al., 2015; Willacker et al., 2019).

On the other hand, although the majority of neglect cases are due to lesions in the right supramarginal gyrus, TPJ, inferior parietal lobule, and superior / middle temporal cortex (Dieterich and Brandt, 2018; Karnath and Rorden, 2012; Lunven and Bartolomeo, 2017; Molenberghs et al., 2012) - regions that presented right lateralized structural connectivity in the current study - there is also evidence that damage to the left insula can also result in neglect (Suchan and Karnath, 2011). The patterns of brain lesions reported in the ‘pusher’ syndrome are also consistent with the hypothesis that an apparently ‘unitary’ clinical syndrome can be caused by lesions in different cortical regions and hemispheres (Baier et al., 2012a; Dieterich and Brandt, 2018; Ticini et al., 2009). Interestingly, functional resting state data on individuals with sub-clinical agoraphobia showed lower connectivity, relative to controls, within a left lateralized network that included insular-somatosensory-motor pathways similar to the OP2 connections outlined here (Indovina et al., 2019). A study assessing cortical folding on patients with persistent postural-perceptual dizziness gave further support to this left-right specialization (Nigro et al., 2019), by showing lower cortical folding in patients vs healthy controls, more pronounced in right than left supramarginal and posterior superior temporal gyri, while in left than right PIC.

### 4.5 Limitations

Diffusion MRI tractography has several limitations, including the difficulty to track subcortical pathways and the lack of directional information about the neuronal projections, i.e., the efferent and afferent connections are indistinguishable from each other. However, employment of a high quality and large sample size, highly controlled database as well as state of the as acquisition, preprocessing and analysis methods, speaks towards high robustness of the results we presented in this paper.

Also, it is important to bear in mind that the lateralization of structural connectivity differs from lateralization in functional terms, as the latter reflects higher activity of a region in one hemisphere, i.e. that hemisphere is dominant for a particular function. However, lateralization in structural connectivity may be a key determinant of functional hemispheric specialization, which is likely to rely on anatomical left-right differences in intra- and interhemispheric connectivity patterns (Stephan et al., 2007). Overall, our results showing structural connectivity lateralization to the left of the Broca area (BA 44, 45) and to the right of the hippocampal-retrosplenial-inferior parietal cortex are in agreement with the dominance of the left hemisphere for language functions, and with the right cerebral cortex dominance for visuo-spatial tasks and navigation (Jager and Postma, 2003; Kaski et al., 2016).

One limitation specific to our study is that fiber density was not corrected for the potential bias towards the gyral crown versus sulcus depth seed region position. Indeed, there is a bias for fiber tracking algorithms to terminate preferentially on gyral crowns, rather than the banks of sulci (Schilling et al., 2018). However, this is unlikely to affect the lateralization analysis as this is done by comparing homonym regions in the two hemispheres that, with good approximation, are located in the same position with respect to sulci. In contrast, it could potentially affect the assessment of hubness, as regions in the depth of sulci could show lower hubness than regions in gyral crowns. However, both PIC and OP2 are located in the depth of the Sylvian fissure and show a high degree of hubness in the current study.

## 5 Conclusions

To summarize, we have shown aspects of the structural connectivity pattern of the vestibular cortex in good agreement with the literature about structural and functional connectivity in human and non-human primates, while some aspects of novelty that can nonetheless be explained in the context of these studies.

On one hand we have shown high hubness and right structural connectivity lateralization of the multimodal vestibular network in high-order associative areas that regulate spatial orientation and navigation. On the other hand, we have demonstrated that those vestibular regions that have been reported to be at the ‘core’ of the vestibular system (OP2, PIC, the insula) display left-lateralized structural connectivity. Overall, these findings inform the current models of vestibular function and may provide new insights to understand the complexity and lateralization of the clinical syndromes related to the visuo-vestibular and somatosensory-vestibular control of balance.

## Appendix Supplementary materials

Image, image 1

Image, application 2

## References

[bib0001] Abe H., Kondo T., Oouchida Y., Suzukamo Y., Fujiwara S., Izumi S.-I. (2012). Prevalence and length of recovery of pusher syndrome based on cerebral hemispheric lesion side in patients with acute stroke. Stroke.

[bib0002] Akbarian S., Grüsser O.J., Guldin W.O. (1994). Corticofugal connections between the cerebral cortex and brainstem vestibular nuclei in the macaque monkey. J. Comp. Neurol..

[bib0003] Akbarian S., Grüsser O.-J., Guldin W.O. (1993). Corticofugal projections to the vestibular nuclei in squirrel monkeys: Further evidence of multiple cortical vestibular fields. J. Comp. Neurol..

[bib0004] Akbarian S., Grüsser O.-J., Guldin W.O. (1992). Thalamic connections of the vestibular cortical fields in the squirrel monkey (Saimiri sciureus). J. Comp. Neurol..

[bib0005] Avants B.B., Tustison N.J., Song G., Cook P.A., Klein A., Gee J.C. (2011). A reproducible evaluation of ANTs similarity metric performance in brain image registration. NeuroImage.

[bib0006] Baier B., Janzen J., Müller-Forell W., Fechir M., Müller N., Dieterich M. (2012). Pusher syndrome: its cortical correlate. J. Neurol.

[bib0007] Baier B., Suchan J., Karnath H.-O., Dieterich M. (2012). Neural correlates of disturbed perception of verticality. Neurology.

[bib0008] Beer A.L., Watanabe T., Ni R., Sasaki Y., Andersen G.J. (2009). 3D surface perception from motion involves a temporal–parietal network. Eur. J. Neurosci..

[bib0009] Betzel R.F., Fukushima M., He Y., Zuo X.-N., Sporns O. (2016). Dynamic fluctuations coincide with periods of high and low modularity in resting-state functional brain networks. NeuroImage.

[bib0010] Bottini G., Sterzi R., Paulesu E., Vallar G., Cappa S.F., Erminio F., Passingham R.E., Frith C.D., Frackowiak R.S. (1994). Identification of the central vestibular projections in man: a positron emission tomography activation study. Exp. Brain Res. Exp. Hirnforsch. Expérimentation Cérébrale.

[bib0011] Brandt T., Dieterich M. (2015). Does the vestibular system determine the lateralization of brain functions?. J. Neurol.

[bib0012] Brandt T., Strupp M., Dieterich M. (2014). Towards a concept of disorders of “higher vestibular function.”. Front. Integr. Neurosci.

[bib0013] Britten K.H. (2008). Mechanisms of self-motion perception. Annu. Rev. Neurosci..

[bib0014] Capozzoli N.J. (1999). Why do we speak with the left hemisphere?. Med. Hypotheses.

[bib0015] Cardin V., Smith A.T. (2010). Sensitivity of human visual and vestibular cortical regions to egomotion-compatible visual stimulation. Cereb. Cortex N. Y. N.

[bib0016] Caruyer E., Lenglet C., Sapiro G., Deriche R. (2013). Design of multishell sampling schemes with uniform coverage in diffusion MRI. Magn. Reson. Med..

[bib0017] Chen A., DeAngelis G.C., Angelaki D.E. (2011). Representation of vestibular and visual cues to self-motion in ventral intraparietal cortex. J. Neurosci. Off. J. Soc. Neurosci..

[bib0018] Chen A., DeAngelis G.C., Angelaki D.E. (2011). Convergence of vestibular and visual self-motion signals in an area of the posterior sylvian fissure. J. Neurosci. Off. J. Soc. Neurosci..

[bib0019] Chen A., DeAngelis G.C., Angelaki D.E. (2010). Macaque parieto-insular vestibular cortex: responses to self-motion and optic flow. J. Neurosci. Off. J. Soc. Neurosci..

[bib0020] Devantier L., Hansen A.K., Mølby-Henriksen J.-J., Christensen C.B., Pedersen M., Hansen K.V., Magnusson M., Ovesen T., Borghammer P. (2020). Positron emission tomography visualized stimulation of the vestibular organ is localized in Heschl's gyrus. Hum. Brain Mapp..

[bib0021] Dieterich M., Bartenstein P., Spiegel S., Bense S., Schwaiger M., Brandt T. (2005). Thalamic infarctions cause side-specific suppression of vestibular cortex activations. Brain J. Neurol..

[bib0022] Dieterich M., Bense S., Lutz S., Drzezga A., Stephan T., Bartenstein P., Brandt T. (2003). Dominance for vestibular cortical function in the non-dominant hemisphere. Cereb. Cortex N. Y. N.

[bib0023] Dieterich M., Brandt T. (2018). Global orientation in space and the lateralization of brain functions. Curr. Opin. Neurol..

[bib0024] Dieterich M., Kirsch V., Brandt T. (2017). Right-sided dominance of the bilateral vestibular system in the upper brainstem and thalamus. J. Neurol..

[bib0025] Dronkers N.F., Knight R.T. (1989). Right-sided neglect in a left-hander: Evidence for reversed hemispheric specialization of attention capacity. Neuropsychologia.

[bib0026] Eickhoff S.B., Schleicher A., Zilles K., Amunts K. (2006). The human parietal operculum. I. Cytoarchitectonic mapping of subdivisions. Cereb. Cortex N. Y. N.

[bib0027] Eickhoff S.B., Stephan K.E., Mohlberg H., Grefkes C., Fink G.R., Amunts K., Zilles K. (2005). A new SPM toolbox for combining probabilistic cytoarchitectonic maps and functional imaging data. NeuroImage.

[bib0028] Fan L., Li H., Zhuo J., Zhang Y., Wang J., Chen L., Yang Z., Chu C., Xie S., Laird A.R., Fox P.T., Eickhoff S.B., Yu C., Jiang T. (2016). The human brainnetome atlas: a new brain atlas based on connectional architecture. Cereb. Cortex N. Y. N.

[bib0029] Fasold O., von Brevern M., Kuhberg M., Ploner C.J., Villringer A., Lempert T., Wenzel R. (2002). Human vestibular cortex as identified with caloric stimulation in functional magnetic resonance imaging. NeuroImage.

[bib0030] Frank S.M., Baumann O., Mattingley J.B., Greenlee M.W. (2014). Vestibular and visual responses in human posterior insular cortex. J. Neurophysiol..

[bib0031] Frank S.M., Greenlee M.W. (2018). The parieto-insular vestibular cortex in humans: more than a single area?. J. Neurophysiol.

[bib0032] Frank S.M., Wirth A.M., Greenlee M.W. (2016). Visual-vestibular processing in the human sylvian fissure. J. Neurophysiol.

[bib0033] Gallivan J.P., Cant J.S., Goodale M.A., Flanagan J.R. (2014). Representation of object weight in human ventral visual cortex. Curr. Biol. CB.

[bib0034] Glasser M.F., Coalson T.S., Robinson E.C., Hacker C.D., Harwell J., Yacoub E., Ugurbil K., Andersson J., Beckmann C.F., Jenkinson M., Smith S.M., Van Essen D.C. (2016). A multi-modal parcellation of human cerebral cortex. Nature.

[bib0035] Grosbras M.-H., Lobel E., Van de Moortele P.-F., LeBihan D., Berthoz A. (1999). An anatomical landmark for the supplementary eye fields in human revealed with functional magnetic resonance imaging. Cereb. Cortex.

[bib0036] Guimerà R., Amaral L.A.N. (2005). Cartography of complex networks: modules and universal roles. J. Stat. Mech..

[bib0037] Guldin W.O., Akbarian S., Grüsser O.J. (1992). Cortico-cortical connections and cytoarchitectonics of the primate vestibular cortex: a study in squirrel monkeys (Saimiri sciureus). J. Comp. Neurol..

[bib0038] Guldin W.O., Grüsser O.J. (1998). Is there a vestibular cortex?. Trends Neurosci.

[bib0039] Hüfner K., Strupp M., Smith P., Brandt T., Jahn K. (2011). Spatial separation of visual and vestibular processing in the human hippocampal formation. Ann. N. Y. Acad. Sci..

[bib0040] Indovina I., Conti A., Lacquaniti F., Staab J.P., Passamonti L., Toschi N. (2019). Lower Functional Connectivity in Vestibular-Limbic Networks in Individuals With Subclinical Agoraphobia. Front. Neurol..

[bib0041] Indovina I., Maffei V., Bosco G., Zago M., Macaluso E., Lacquaniti F. (2005). Representation of visual gravitational motion in the human vestibular cortex. Science.

[bib0042] Indovina I., Maffei V., Mazzarella E., Sulpizio V., Galati G., Lacquaniti F. (2016). Path integration in 3D from visual motion cues: a human fMRI study. NeuroImage..

[bib0043] Indovina I., Maffei V., Pauwels K., Macaluso E., Orban G.A., Lacquaniti F. (2013). Simulated self-motion in a visual gravity field: Sensitivity to vertical and horizontal heading in the human brain. NeuroImage.

[bib0044] Jager G., Postma A. (2003). On the hemispheric specialization for categorical and coordinate spatial relations: a review of the current evidence. Neuropsychologia.

[bib0045] Janzen J., Schlindwein P., Bense S., Bauermann T., Vucurevic G., Stoeter P., Dieterich M. (2008). Neural correlates of hemispheric dominance and ipsilaterality within the vestibular system. NeuroImage.

[bib0046] Jenkinson M., Beckmann C.F., Behrens T.E.J., Woolrich M.W., Smith S.M. (2012). FSL. NeuroImage.

[bib0047] Jeurissen B., Descoteaux M., Mori S., Leemans A. (2019). Diffusion MRI fiber tractography of the brain. NMR Biomed.

[bib0048] Jeurissen B., Tournier J.-D., Dhollander T., Connelly A., Sijbers J. (2014). Multi-tissue constrained spherical deconvolution for improved analysis of multi-shell diffusion MRI data. NeuroImage.

[bib0049] Karnath H.-O., Dieterich M. (2006). Spatial neglect–a vestibular disorder?. Brain J. Neurol..

[bib0050] Karnath H.-O., Rorden C. (2012). The anatomy of spatial neglect. Neuropsychologia.

[bib0051] Karolis V.R., Corbetta M., Thiebaut de Schotten M. (2019). The architecture of functional lateralisation and its relationship to callosal connectivity in the human brain. Nat. Commun..

[bib0052] Kaski D., Quadir S., Nigmatullina Y., Malhotra P.A., Bronstein A.M., Seemungal B.M. (2016). Temporoparietal encoding of space and time during vestibular-guided orientation. Brain.

[bib0053] Kheradmand A., Winnick A. (2017). Perception of Upright: Multisensory Convergence and the Role of Temporo-Parietal Cortex. Front. Neurol..

[bib0054] Kirsch V., Boegle R., Keeser D., Kierig E., Ertl-Wagner B., Brandt T., Dieterich M. (2018). Handedness-dependent functional organizational patterns within the bilateral vestibular cortical network revealed by fMRI connectivity based parcellation. NeuroImage.

[bib0055] Kirsch V., Keeser D., Hergenroeder T., Erat O., Ertl-Wagner B., Brandt T., Dieterich M. (2016). Structural and functional connectivity mapping of the vestibular circuitry from human brainstem to cortex. Brain Struct. Funct..

[bib0056] Kravitz D.J., Saleem K.S., Baker C.I., Mishkin M. (2011). A new neural framework for visuospatial processing. Nat. Rev. Neurosci..

[bib0057] Lacquaniti F., Bosco G., Gravano S., Indovina I., La Scaleia B., Maffei V., Zago M. (2014). Multisensory integration and internal models for sensing gravity effects in primates. BioMed Res. Int..

[bib0058] Lacquaniti F., Bosco G., Indovina I., La Scaleia B., Maffei V., Moscatelli A., Zago M. (2013). Visual gravitational motion and the vestibular system in humans. Front. Integr. Neurosci..

[bib0059] Lancichinetti A., Fortunato S. (2012). Consensus clustering in complex networks. Sci. Rep..

[bib0060] Latora V., Marchiori M. (2001). Efficient behavior of small-world networks. Phys. Rev. Lett..

[bib0061] Lopez C., Blanke O. (2011). The thalamocortical vestibular system in animals and humans. Brain Res. Rev..

[bib0062] Lopez C., Blanke O., Mast F.W. (2012). The human vestibular cortex revealed by coordinate-based activation likelihood estimation meta-analysis. Neuroscience.

[bib0063] Lunven M., Bartolomeo P. (2017). Attention and spatial cognition: neural and anatomical substrates of visual neglect. Ann. Phys. Rehabil. Med..

[bib0064] Maffei V., Indovina I., Macaluso E., Ivanenko Y.P., A Orban G., Lacquaniti F. (2015). Visual gravity cues in the interpretation of biological movements: neural correlates in humans. NeuroImage.

[bib0065] Maffei V., Mazzarella E., Piras F., Spalletta G., Caltagirone C., Lacquaniti F., Daprati E. (2016). Processing of visual gravitational motion in the peri-sylvian cortex: Evidence from brain-damaged patients. Cortex J. Devoted Study Nerv. Syst. Behav..

[bib0066] Maguire E.A., Burgess N., Donnett J.G., Frackowiak R.S., Frith C.D., O'Keefe J. (1998). Knowing where and getting there: a human navigation network. Science.

[bib0067] Mazzola L., Faillenot I., Barral F.-G., Mauguière F., Peyron R. (2012). Spatial segregation of somato-sensory and pain activations in the human operculo-insular cortex. NeuroImage.

[bib0068] Mazzola L., Lopez C., Faillenot I., Chouchou F., Mauguière F., Isnard J. (2014). Vestibular responses to direct stimulation of the human insular cortex. Ann. Neurol..

[bib0069] Molenberghs P., Sale M.V., Mattingley J.B. (2012). Is there a critical lesion site for unilateral spatial neglect? A meta-analysis using activation likelihood estimation. Front. Hum. Neurosci..

[bib0070] Nigro S., Indovina I., Riccelli R., Chiarella G., Petrolo C., Lacquaniti F., Staab J.P., Passamonti L. (2019). Reduced cortical folding in multi-modal vestibular regions in persistent postural perceptual dizziness. Brain Imaging Behav.

[bib0071] Oldfield R.C. (1971). The assessment and analysis of handedness: the Edinburgh inventory. Neuropsychologia.

[bib0072] Riccelli R., Indovina I., Staab J.P., Nigro S., Augimeri A., Lacquaniti F., Passamonti L. (2017). Neuroticism modulates brain visuo-vestibular and anxiety systems during a virtual rollercoaster task. Hum. Brain Mapp..

[bib0073] Roberts J.A., Perry A., Roberts G., Mitchell P.B., Breakspear M. (2017). Consistency-based thresholding of the human connectome. NeuroImage.

[bib0074] Rousseau C., Fautrelle L., Papaxanthis C., Fadiga L., Pozzo T., White O. (2016). Direction-dependent activation of the insular cortex during vertical and horizontal hand movements. Neuroscience.

[bib0075] Rousseaux M., Braem B., Honoré J., Saj A. (2015). An anatomical and psychophysical comparison of subjective verticals in patients with right brain damage. Cortex J. Devoted Study Nerv. Syst. Behav..

[bib0076] Rubinov M., Sporns O. (2010). Complex network measures of brain connectivity: uses and interpretations. NeuroImage.

[bib0077] Scharoun S.M., Bryden P.J. (2014). Hand preference, performance abilities, and hand selection in children. Front. Psychol..

[bib0078] Schilling K., Gao Y., Janve V., Stepniewska I., Landman B.A., Anderson A.W. (2018). Confirmation of a gyral bias in diffusion MRI fiber tractography. Hum. Brain Mapp..

[bib0079] Schlindwein P., Mueller M., Bauermann T., Brandt T., Stoeter P., Dieterich M. (2008). Cortical representation of saccular vestibular stimulation: VEMPs in fMRI. NeuroImage.

[bib0080] Shinder M.E., Newlands S.D. (2014). Sensory convergence in the parieto-insular vestibular cortex. J. Neurophysiol..

[bib0081] Smith R.E., Tournier J.-D., Calamante F., Connelly A. (2013). SIFT: Spherical-deconvolution informed filtering of tractograms. NeuroImage.

[bib0082] Sotiropoulos S.N., Jbabdi S., Xu J., Andersson J.L., Moeller S., Auerbach E.J., Glasser M.F., Hernandez M., Sapiro G., Jenkinson M., Feinberg D.A., Yacoub E., Lenglet C., Van Essen D.C., Ugurbil K., Behrens T.E.J. (2013). Advances in diffusion MRI acquisition and processing in the human connectome project. NeuroImage, Mapping the Connectome.

[bib0083] Stephan K.E., Fink G.R., Marshall J.C. (2007). Mechanisms of hemispheric specialization: insights from analyses of connectivity. Neuropsychologia.

[bib0084] Stephan T., Deutschländer A., Nolte A., Schneider E., Wiesmann M., Brandt T., Dieterich M. (2005). Functional MRI of galvanic vestibular stimulation with alternating currents at different frequencies. NeuroImage.

[bib0085] Suchan J., Karnath H.-O. (2011). Spatial orienting by left hemisphere language areas: a relict from the past?. Brain.

[bib0086] Sunaert S., Van Hecke P., Marchal G., Orban G.A. (1999). Motion-responsive regions of the human brain. Exp. Brain Res. Exp. Hirnforsch. Expérimentation Cérébrale.

[bib0087] Suzuki M., Kitano H., Ito R., Kitanishi T., Yazawa Y., Ogawa T., Shiino A., Kitajima K. (2001). Cortical and subcortical vestibular response to caloric stimulation detected by functional magnetic resonance imaging. Brain Res. Cogn. Brain Res..

[bib0088] Ticini L.F., Klose U., Nägele T., Karnath H.-O. (2009). Perfusion imaging in pusher syndrome to investigate the neural substrates involved in controlling upright body position. PLOS ONE.

[bib0089] Tournier J.-D., Calamante F., Connelly A. (2007). Robust determination of the fibre orientation distribution in diffusion MRI: non-negativity constrained super-resolved spherical deconvolution. NeuroImage.

[bib0090] Tournier J.-D., Smith R., Raffelt D., Tabbara R., Dhollander T., Pietsch M., Christiaens D., Jeurissen B., Yeh C.-H., Connelly A. (2019). MRtrix3: a fast, flexible and open software framework for medical image processing and visualisation. NeuroImage.

[bib0091] Vanderploeg R.D. (1986). Left-handedness and variant patterns of cerebral organization: a case study. Arch. Clin. Neuropsychol..

[bib0092] Ventre-Dominey J. (2014). Vestibular function in the temporal and parietal cortex: distinct velocity and inertial processing pathways. Front. Integr. Neurosci..

[bib0093] Vitte E., Derosier C., Caritu Y., Berthoz A., Hasboun D., Soulié D. (1996). Activation of the hippocampal formation by vestibular stimulation: a functional magnetic resonance imaging study. Exp. Brain Res. Exp. Hirnforsch. Expérimentation Cérébrale.

[bib0094] Willacker L., Dowsett J., Dieterich M., Taylor P.C.J. (2019). Egocentric processing in the roll plane and dorsal parietal cortex: A TMS-ERP study of the subjective visual vertical. Neuropsychologia.

[bib0095] Willats L., Raffelt D., Smith R.E., Tournier J.-D., Connelly A., Calamante F. (2014). Quantification of track-weighted imaging (TWI): characterisation of within-subject reproducibility and between-subject variability. NeuroImage.

[bib0096] Wirth A.M., Frank S.M., Greenlee M.W., Beer A.L. (2018). White matter connectivity of the visual-vestibular cortex examined by diffusion-weighted imaging. Brain Connect.

[bib0097] zu Eulenburg P., Caspers S., Roski C., Eickhoff S.B. (2012). Meta-analytical definition and functional connectivity of the human vestibular cortex. NeuroImage.

