# Supplementary figures and images for: Structural connectome and connectivity lateralization of the multimodal vestibular cortical network
Source: Neuroimage. 2020 Nov 15;222:117247. doi: 10.1016/j.neuroimage.2020.117247 (PMC7779422; doi:10.1016/j.neuroimage.2020.117247)

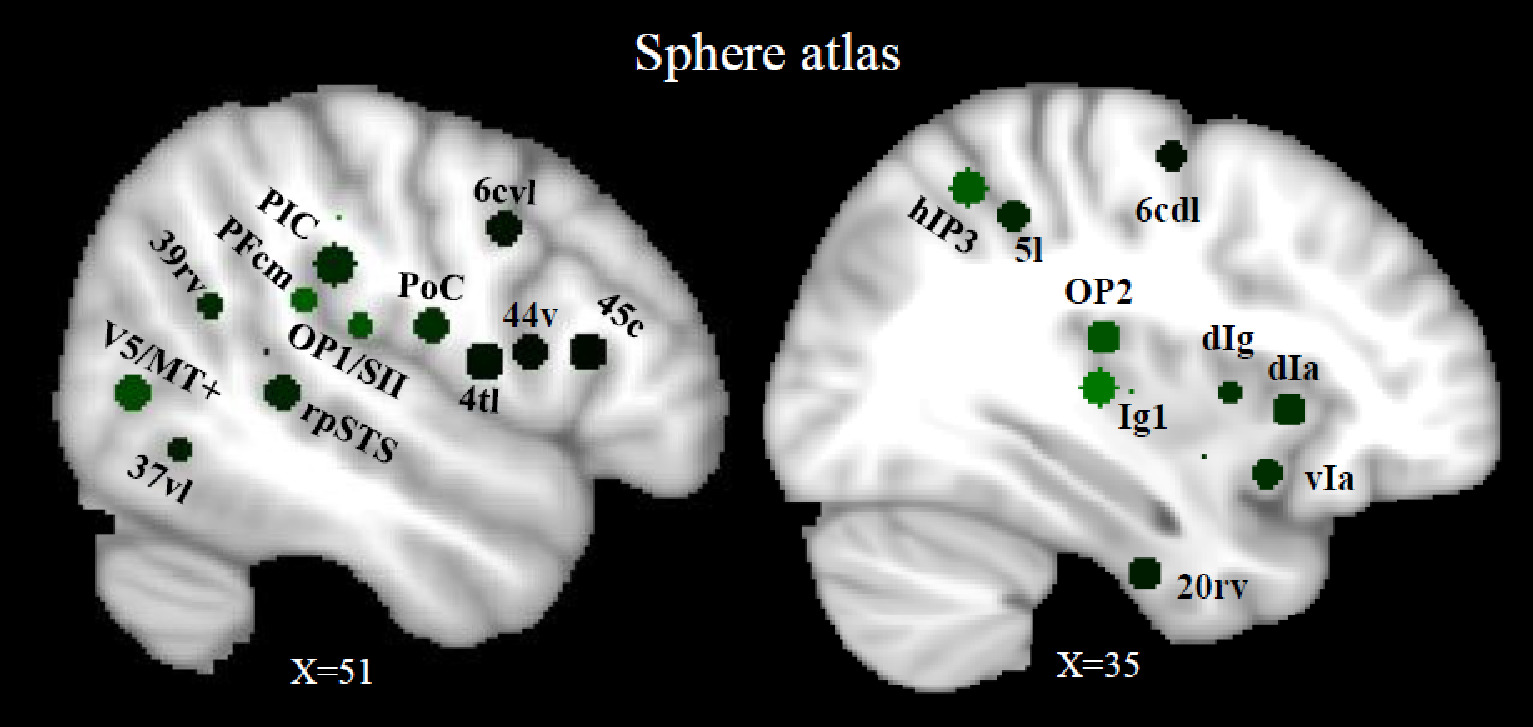

Supplement: Supplementary file 1 [file mmc1.jpg]
