# Supplementary material for: Structural connectome and connectivity lateralization of the multimodal vestibular cortical network
Source: Neuroimage. 2020 Nov 15;222:117247. doi: 10.1016/j.neuroimage.2020.117247 (PMC7779422; doi:10.1016/j.neuroimage.2020.117247)
Supplement: Supplementary file 2 [file mmc2.docx]

Supplementary Table1.

| **Connectivity of PIC Right vs connectivity of OP2 Right**   \| **ipsi** \| **conn** \| \|  \| **contra** \| **conn** \| \| --- \| --- \| --- \| --- \| --- \| --- \| \| PFcm (IPL) \| \| 260.86 \|  \| 123tru \| 0.96 \| \| PF (IPL) \| \| 163.44 \|  \| 5L (SPL) \| 0.73 \| \| OP1 (SII) \| \| 142.07 \|  \| 5m \| 0.54 \| \| PFt (IPL) \| \| 141.79 \|  \| Thal Parietal \| 0.42 \| \| PFop (IPL) \| \| 131.52 \|  \| PFop (IPL) \| 0.38 \| \| 40rd \| \| 68.42 \|  \| 5M (SPL) \| 0.36 \| \| 40c \| \| 39.59 \|  \| 7pc \| 0.28 \| \| 2 \| \| 18.88 \|  \| 7m \| 0.24 \| \| 1 \| \| 4.90 \|  \| Thal Somatosens \| 0.23 \| \| hIP2 (IPS) \| \| 4.79 \|  \| 123ll \| 0.22 \| \| PFm (IPL) \| \| 2.87 \|  \| PPtha \| 0.19 \| \| 3b \| \| 2.78 \|  \| 7r \| 0.17 \| \| cpSTS \| 2.68 \| \|  \| 4a \| 0.17 \| \| Thal Parietal \| 2.47 \| \|  \| Stha \| 0.16 \| \| 44 \| 2.05 \| \|  \| cTtha \| 0.14 \| \| PGa (IPL) \| 1.25 \| \|  \| rHipp \| 0.14 \| \| 3a \| 1.13 \| \|  \| 7P (SPL) \| 0.13 \| \| 2 \| 1.10 \| \|  \| Ig1 \| 0.12 \| \| 5L (SPL) \| 0.96 \| \|  \| PF (IPL) \| 0.12 \| \| 5l \| 0.93 \| \|  \| PFt (IPL) \| 0.12 \| \| 44v \| 0.67 \| \|  \| 5Ci (SPL) \| 0.12 \| \| 4hf \| 0.64 \| \|  \| Thal Temporal \| 0.11 \| \| 44d \| 0.60 \| \|  \| 7PC (SPL) \| 0.11 \| \| 45r \| 0.59 \| \|  \| PIC \| 0.10 \| \| Thal Somatosens \| 0.59 \| \|  \| Ig2 \| 0.09 \| \| 21c \| 0.58 \| \|  \| 4t \| 0.08 \| \| 37dl \| 0.57 \| \|  \| 40rd \| 0.08 \| \| 5m \| 0.57 \| \|  \| OP1 (SII) \| 0.07 \| \| Stha \| 0.54 \| \|  \| 7A (SPL) \| 0.07 \| \| IFS \| 0.49 \| \|  \| Thal Motor \| 0.06 \| \| 45 \| 0.47 \| \|  \| TE 10 \| 0.06 \| \| 123ulhf \| 0.44 \| \|  \| 2 \| 0.06 \| \| rHipp \| 0.40 \| \|  \| 33 \| 0.05 \| \| hIP1 (IPS) \| 0.39 \| \|  \| 1 \| 0.05 \| \| 5M (SPL) \| 0.38 \| \|  \| PFcm (IPL) \| 0.05 \| \| rpSTS \| 0.35 \| \|  \| 4142 \| 0.04 \| \| aSTS \| 0.35 \| \|  \| 23v \| 0.04 \| \| Thal Motor \| 0.35 \| \|  \| TE 3 \| 0.04 \| \| Thal Temporal \| 0.31 \| \|  \| 44v \| 0.04 \| \| PPtha \| 0.30 \| \|  \| 123ulhf \| 0.04 \| \| 20cl \| 0.30 \| \|  \| 4ll \| 0.04 \| \| 39rv \| 0.30 \| \|  \| 3b \| 0.04 \| \| 45c \| 0.29 \| \|  \| 5l \| 0.03 \| \| hIP3 (IPS) \| 0.25 \| \|  \| G \| 0.03 \| \| 946v \| 0.24 \| \|  \|  \|  \| \| 123ll \| 0.22 \| \|  \|  \|  \| \| Otha \| 0.22 \| \|  \|  \|  \| \| 6cvl \| 0.21 \| \|  \|  \|  \| \| 7m \| 0.20 \| \|  \|  \|  \| \| IFJ \| 0.20 \| \|  \|  \|  \| \| 5Ci (SPL) \| 0.19 \| \|  \|  \|  \| \| 10l \| 0.14 \| \|  \|  \|  \| \| 37elv \| 0.13 \| \|  \|  \|  \| \| 37vl \| 0.12 \| \|  \|  \|  \| \| PGp (IPL) \| 0.12 \| \|  \|  \|  \| \| 1247o \| 0.12 \| \|  \|  \|  \| \| 8vl \| 0.10 \| \|  \|  \|  \| \| 6m \| 0.07 \| \|  \|  \|  \| \| Thal Premotor \| 0.07 \| \|  \|  \|  \| \| 20iv \| 0.07 \| \|  \|  \|  \| \| cTtha \| 0.06 \| \|  \|  \|  \| \| Thal Prefrontal \| 0.05 \| \|  \|  \|  \|   **Connectivity of OP2 Right vs connectivity of PIC Right (there are only ipsilateral connections)** |  |
| --- | --- | --- | --- | --- | --- | --- | --- | --- | --- | --- | --- | --- | --- | --- | --- | --- | --- | --- | --- | --- | --- | --- | --- | --- | --- | --- | --- | --- | --- | --- | --- | --- | --- | --- | --- | --- | --- | --- | --- | --- | --- | --- | --- | --- | --- | --- | --- | --- | --- | --- | --- | --- | --- | --- | --- | --- | --- | --- | --- | --- | --- | --- | --- | --- | --- | --- | --- | --- | --- | --- | --- | --- | --- | --- | --- | --- | --- | --- | --- | --- | --- | --- | --- | --- | --- | --- | --- | --- | --- | --- | --- | --- | --- | --- | --- | --- | --- | --- | --- | --- | --- | --- | --- | --- | --- | --- | --- | --- | --- | --- | --- | --- | --- | --- | --- | --- | --- | --- | --- | --- | --- | --- | --- | --- | --- | --- | --- | --- | --- | --- | --- | --- | --- | --- | --- | --- | --- | --- | --- | --- | --- | --- | --- | --- | --- | --- | --- | --- | --- | --- | --- | --- | --- | --- | --- | --- | --- | --- | --- | --- | --- | --- | --- | --- | --- | --- | --- | --- | --- | --- | --- | --- | --- | --- | --- | --- | --- | --- | --- | --- | --- | --- | --- | --- | --- | --- | --- | --- | --- | --- | --- | --- | --- | --- | --- | --- | --- | --- | --- | --- | --- | --- | --- | --- | --- | --- | --- | --- | --- | --- | --- | --- | --- | --- | --- | --- | --- | --- | --- | --- | --- | --- | --- | --- | --- | --- | --- | --- | --- | --- | --- | --- | --- | --- | --- | --- | --- | --- | --- | --- | --- | --- | --- | --- | --- | --- | --- | --- | --- | --- | --- | --- | --- | --- | --- | --- | --- | --- | --- | --- | --- | --- | --- | --- | --- | --- | --- | --- | --- | --- | --- | --- | --- | --- | --- | --- | --- | --- | --- | --- | --- | --- | --- | --- | --- | --- | --- | --- | --- | --- | --- | --- | --- | --- | --- | --- | --- | --- | --- | --- | --- | --- | --- | --- | --- | --- | --- | --- | --- | --- | --- | --- | --- | --- | --- | --- | --- | --- | --- | --- | --- | --- | --- | --- | --- | --- | --- | --- | --- | --- | --- | --- | --- | --- | --- | --- | --- | --- | --- | --- | --- | --- | --- | --- | --- | --- | --- | --- | --- | --- | --- | --- | --- | --- | --- | --- | --- | --- | --- | --- | --- | --- | --- | --- | --- | --- | --- | --- | --- | --- | --- | --- | --- | --- | --- | --- | --- | --- | --- |

**Ipsi conn**

| Ig2 | 154.0227 |
| --- | --- |
| G | 131.2355 |
| OP3 (VS) | 90.9937 |
| TE 1.1 | 78.2065 |
| Ig1 | 73.4937 |
| dIg | 21.0919 |
| TE 1.0 | 10.922 |
| 41/42 | 8.7746 |
| dlPu | 7.7721 |
| 123tonIa | 6.17 |
| 22c | 4.874 |
| 123tru | 2.7355 |
| dId | 2.1561 |
| TE 1.2 | 2.0176 |
| 7PC (SPL) | 0.9345 |

**Connectivity of PIC Left vs connectivity of OP2 Left**

| ipsi | conn |  | contra | conn |
| --- | --- | --- | --- | --- |
| PFcm (IPL) | 578.81 |  | 5L (SPL) | 1.56 |
| PFop (IPL) | 328.78 |  | Thal Parietal | 1.15 |
| PF (IPL) | 66.25 |  | 5m | 0.91 |
| 4142 | 56.91 |  | 7pc | 0.89 |
| PFt (IPL) | 32.36 |  | 7m | 0.62 |
| 22c | 29.76 |  | 5M (SPL) | 0.57 |
| 40c | 16.82 |  | 40rd | 0.53 |
| Thal Parietal | 6.00 |  | 7PC (SPL) | 0.48 |
| 21c | 5.95 |  | 123tru | 0.48 |
| 44 | 5.06 |  | 7r | 0.43 |
| rpSTS | 4.39 |  | 4a | 0.37 |
| 44v | 4.08 |  | PF (IPL) | 0.33 |
| rHipp | 3.66 |  | Otha | 0.26 |
| aSTS | 3.18 |  | 5Ci (SPL) | 0.26 |
| 45 | 3.11 |  | Stha | 0.25 |
| PPtha | 3.04 |  | 123ll | 0.24 |
| 20cl | 2.93 |  | 22c | 0.24 |
| 40rd | 2.91 |  | PPtha | 0.24 |
| PFm (IPL) | 2.64 |  | 40c | 0.22 |
| 44d | 2.47 |  | Ig1 | 0.20 |
| hIP2 (IPS) | 2.38 |  | 7 (SPL) | 0.17 |
| 37dl | 2.18 |  | rHipp | 0.17 |
| cpSTS | 1.63 |  | 2 | 0.16 |
| cTtha | 1.54 |  | PFt (IPL) | 0.15 |
| Stha | 1.46 |  | 7P (SPL) | 0.15 |
| Thal Somatosensory | 1.45 |  | PFcm (IPL) | 0.14 |
| 5m | 1.38 |  | Thal Temporal | 0.14 |
| 20il | 1.38 |  | Thal Somatosensory | 0.13 |
| 21r | 1.25 |  | hIP2 (IPS) | 0.13 |
| 37vl | 1.19 |  | PFop (IPL) | 0.13 |
| 45c | 1.14 |  | Thal Visual | 0.13 |
| Thal Visual | 1.05 |  | 4t | 0.13 |
| Thal Temporal | 1.04 |  | TE 11 | 0.12 |
| 20r | 0.88 |  | 4142 | 0.11 |
| 7m | 0.78 |  | TE 3 | 0.10 |
| 20cv | 0.78 |  | PIC | 0.10 |
| IFJ | 0.75 |  | 31 | 0.10 |
| 45r | 0.68 |  | TE 10 | 0.09 |
| vIdvIg | 0.63 |  | Ig2 | 0.09 |
| IFS | 0.57 |  | 2 | 0.09 |
| 7P (SPL) | 0.52 |  | 23d | 0.08 |
| 23d | 0.50 |  | OP1 (SII) | 0.07 |
| 946v | 0.37 |  | 7M (SPL) | 0.07 |
| vIa | 0.33 |  | PFm (IPL) | 0.07 |
| 6cvl | 0.32 |  | cpSTS | 0.07 |
| 38l | 0.28 |  | 1 | 0.06 |
| mygdala (Str) | 0.27 |  | 22r | 0.06 |
| 37elv | 0.27 |  | Thal Motor | 0.06 |
| Otha | 0.23 |  | cTtha | 0.05 |
| Subiculum | 0.20 |  | TE 12 | 0.05 |
| 23v | 0.18 |  | G | 0.05 |
| PGa (IPL) | 0.17 |  | 7ip | 0.04 |
| 10l | 0.16 |  | PGa (IPL) | 0.03 |
| lmyg | 0.14 |  | mPFtha | 0.02 |
| 24rv | 0.13 |  |  |  |
| mygdala (CM) | 0.13 |  |  |  |
| 39rv | 0.13 |  |  |  |
| 20iv | 0.10 |  |  |  |
| 31 | 0.09 |  |  |  |
| hOc4v (V4(v)) | 0.06 |  |  |  |
| BF (Ch 4) | 0.06 |  |  |  |
| 38m | 0.06 |  |  |  |
| 20rv | 0.06 |  |  |  |
| cHipp | 0.05 |  |  |  |
| DG (Hippocampus) | 0.03 |  |  |  |

**Connectivity of OP2 Left vs connectivity of PIC Left**

| ipsi | conn |
| --- | --- |
| G | 449.93 |
| Ig1 | 403.45 |
| TE 11 | 199.96 |
| Ig2 | 154.47 |
| TE 10 | 147.99 |
| OP3 (VS) | 96.92 |
| dIg | 27.15 |
| TE 12 | 17.61 |
| 7pc | 13.73 |
| 123tonIa | 12.52 |
| TE10TE12_ | 11.05 |
| 123tru | 10.69 |
| OP1 (SII) | 9.89 |
| OP4 (PV) | 9.82 |
| 7PC (SPL) | 9.43 |
| 4tl | 8.83 |
| 1 | 4.65 |
| 2 | 3.88 |
| Id1 | 3.15 |
| 5l | 2.22 |
| 5L (SPL) | 1.68 |
| dId | 1.47 |
| 7ip | 1.19 |
| 3a | 1.13 |
| 4a | 1.13 |
| 4ul | 0.99 |
| 3b | 0.76 |
| 4p | 0.54 |
| 6vl | 0.51 |
| dlPu | 0.42 |
| hIP3 (IPS) | 0.31 |
| 6cdl | 0.17 |
| hIP1 (IPS) | 0.13 |
| 2 | 0.09 |

**Supplementary Table2.** To show that the laterality analysis does not substantially change with or without normalization for streamline length we calculated the Structural connectivity Lateralization index (L) also after normalizing for streamline length (see Table 2 in the text for the corresponding analysis without normalization). Lateralization length is the total number of above threshold connections of each area in both hemispheres.

| **Area** | **Left L** | **L length** | **pBonf** |  |  | **Right L** | **L length** | **pBonf** |
| --- | --- | --- | --- | --- | --- | --- | --- | --- |
| 35/36c | -1.00 | 34 | 0.00 |  | hOc5 (V5MT) | 1.00 | 31 | 0.00 |
| 28/34 | -1.00 | 45 | 0.00 |  | 37mv | 0.42 | 50 | 0.00 |
| 5l | -1.00 | 34 | 0.00 |  | 33 | 0.35 | 206 | 0.00 |
| 7pc | -1.00 | 68 | 0.00 |  | 14m | 0.31 | 84 | 0.00 |
| 40c | -1.00 | 80 | 0.00 |  | TE10TE12_ | 0.31 | 159 | 0.00 |
| OP2 (PIVC) | -1.00 | 56 | 0.00 |  | 24rv | 0.29 | 204 | 0.00 |
| 25 | -1.00 | 17 | 1.21 |  | 123ll | 0.28 | 170 | 0.00 |
| Ig1 | -0.60 | 155 | 0.00 |  | C2 (Hippocampus) | 0.28 | 25 | 0.03 |
| vIa | -0.50 | 159 | 0.00 |  | 4142 | 0.27 | 208 | 0.00 |
| dIa | -0.50 | 173 | 0.00 |  | hIP2 (IPS) | 0.26 | 150 | 0.00 |
| 40rv | -0.50 | 146 | 0.00 |  | s32 | 0.25 | 70 | 0.01 |
| 7ip | -0.48 | 97 | 0.00 |  | hIP1 (IPS) | 0.20 | 155 | 0.00 |
| dIg | -0.46 | 151 | 0.00 |  | aSTS | 0.20 | 225 | 0.00 |
| dId | -0.44 | 211 | 0.00 |  | 10m | 0.18 | 144 | 0.00 |
| Fo3 | -0.43 | 50 | 0.02 |  | cpSTS | 0.16 | 162 | 0.00 |
| G | -0.42 | 129 | 0.00 |  | PFt (IPL) | 0.16 | 203 | 0.00 |
| 11l | -0.40 | 30 | 0.00 |  | 23d | 0.14 | 311 | 0.00 |
| 44v | -0.38 | 134 | 0.00 |  | 20iv | 0.14 | 121 | 0.00 |
| Ig2 | -0.38 | 212 | 0.00 |  | 23v | 0.14 | 328 | 0.00 |
| TL | -0.36 | 62 | 0.00 |  | hIP3 (IPS) | 0.14 | 220 | 0.00 |
| 37lv | -0.34 | 89 | 0.00 |  | PFcm (IPL) | 0.13 | 177 | 0.00 |
| 5Ci (SPL) | -0.33 | 109 | 0.00 |  | 4p | 0.13 | 158 | 0.00 |
| rHipp | -0.26 | 295 | 0.00 |  | rpSTS | 0.12 | 156 | 0.00 |
| Subiculum | -0.23 | 218 | 0.00 |  | C1 (Hippocampus) | 0.12 | 142 | 0.00 |
| V5MT+ | -0.20 | 129 | 0.00 |  | 5m | 0.12 | 233 | 0.00 |
| 45c | -0.27 | 115 | 0.00 |  | Lobule IX (Hem) | 0.11 | 119 | 0.00 |
| rHipp | -0.26 | 295 | 0.00 |  | 7PC (SPL) | 0.11 | 186 | 0.00 |
| 20r | -0.25 | 126 | 0.00 |  | Amygdala (Str) | 0.11 | 69 | 0.05 |
| Subiculum | -0.23 | 218 | 0.00 |  | 39c | 0.11 | 190 | 0.00 |
| 10l | -0.21 | 102 | 0.00 |  | 9m | 0.11 | 232 | 0.00 |
| V5MT+ | -0.20 | 129 | 0.00 |  | 2 | 0.10 | 192 | 0.00 |
| TE 10 | -0.19 | 163 | 0.00 |  | 6cdl | 0.10 | 236 | 0.00 |
| 45 | -0.19 | 234 | 0.00 |  | PF (IPL) | 0.09 | 266 | 0.00 |
| 4hf | -0.19 | 217 | 0.00 |  | Lobule VIIa crusII (Hem) | 0.08 | 175 | 0.00 |
| 1247l | -0.18 | 172 | 0.01 |  | Lobule VIIa crusI (Hem) | 0.08 | 182 | 0.00 |
| 946v | -0.17 | 220 | 0.00 |  | dlPu | 0.07 | 354 | 0.00 |
| 38l | -0.17 | 159 | 0.00 |  | Lobule VIIIb (Hem) | 0.07 | 81 | 0.03 |
| OP1 (SII) | -0.17 | 145 | 0.00 |  | PGa (IPL) | 0.05 | 222 | 0.03 |
| 7r | -0.16 | 84 | 0.00 |  | 7 (SPL) | 0.03 | 326 | 0.05 |
| 45r | -0.16 | 155 | 0.00 |  |  |  |  |  |
| IFJ | -0.15 | 222 | 0.00 |  |  |  |  |  |
| 20rv | -0.15 | 165 | 0.00 |  |  |  |  |  |
| BF (Ch 4) | -0.15 | 132 | 0.00 |  |  |  |  |  |
| Ventral Dentate Nucleus | -0.15 | 63 | 0.00 |  |  |  |  |  |
| mOccG | -0.14 | 99 | 0.00 |  |  |  |  |  |
| FG2 | -0.14 | 109 | 0.00 |  |  |  |  |  |
| TE 11 | -0.14 | 167 | 0.00 |  |  |  |  |  |
| OP3 (VS) | -0.14 | 125 | 0.00 |  |  |  |  |  |
| Lobule X (Hem) | -0.14 | 81 | 0.00 |  |  |  |  |  |
| 24cd | -0.13 | 116 | 0.00 |  |  |  |  |  |
| 123ulhf | -0.13 | 138 | 0.00 |  |  |  |  |  |
| hOc4v (V4(v)) | -0.12 | 148 | 0.00 |  |  |  |  |  |
| 44d | -0.12 | 169 | 0.00 |  |  |  |  |  |
| hOc4la | -0.11 | 190 | 0.00 |  |  |  |  |  |
| vIdvIg | -0.11 | 246 | 0.00 |  |  |  |  |  |
| Lobule V (Hem) | -0.10 | 117 | 0.00 |  |  |  |  |  |
| Thal Temporal | -0.10 | 361 | 0.00 |  |  |  |  |  |
| 22r | -0.10 | 158 | 0.00 |  |  |  |  |  |
| 39rv | -0.09 | 142 | 0.01 |  |  |  |  |  |
| 44 | -0.09 | 230 | 0.00 |  |  |  |  |  |
| rLinG | -0.09 | 130 | 0.00 |  |  |  |  |  |
| hOc3v (V3v) | -0.08 | 169 | 0.00 |  |  |  |  |  |
| Thal Prefrontal | -0.08 | 330 | 0.00 |  |  |  |  |  |
| Otha | -0.08 | 114 | 0.04 |  |  |  |  |  |
| 44op | -0.08 | 180 | 0.00 |  |  |  |  |  |
| Lobule I IV (Hem) | -0.08 | 268 | 0.00 |  |  |  |  |  |
| 20cv | -0.07 | 135 | 0.01 |  |  |  |  |  |
| 21r | -0.07 | 181 | 0.00 |  |  |  |  |  |
| FG4 | -0.07 | 125 | 0.01 |  |  |  |  |  |
| 9l | -0.07 | 252 | 0.04 |  |  |  |  |  |
| 6dl | -0.07 | 281 | 0.00 |  |  |  |  |  |
| 946d | -0.06 | 215 | 0.02 |  |  |  |  |  |
| Thal Parietal | -0.06 | 358 | 0.00 |  |  |  |  |  |
| 20il | -0.06 | 162 | 0.00 |  |  |  |  |  |
| 4ul | -0.06 | 204 | 0.01 |  |  |  |  |  |
| 37vl | -0.05 | 168 | 0.02 |  |  |  |  |  |
| hOc2 (V2) | -0.04 | 178 | 0.05 |  |  |  |  |  |
| vmPu | -0.04 | 290 | 0.01 |  |  |  |  |  |
